# Marine Guanidine Alkaloids Inhibit Malaria Parasites Development in *In Vitro*, *In Vivo* and *Ex Vivo* Assays

**DOI:** 10.1021/acsinfecdis.4c00714

**Published:** 2025-04-15

**Authors:** Giovana Rossi Mendes, Anderson L. Noronha, Igor M. R. Moura, Natália Menezes Moreira, Vinícius Bonatto, Camila S. Barbosa, Sarah El Chamy Maluf, Guilherme Eduardo de Souza, Marcelo Rodrigues de Amorim, Anna Caroline Campos Aguiar, Fabio C. Cruz, Amália dos Santos Ferreira, Carolina B. G. Teles, Dhelio B. Pereira, Eduardo Hajdu, Antonio G. Ferreira, Roberto G. S. Berlinck, Rafael Victorio Carvalho Guido

**Affiliations:** † São Carlos Institute of Physics, 117186 University of Sao Paulo, CEP 13563-120 São Carlos, SP, Brazil; ‡ Instituto de Química de São Carlos, 153988 Universidade de São Paulo, CEP 13560-970 São Carlos, SP, Brazil; § Department of Microbiology, Immunology and Parasitology, Federal University of São Paulo, CEP 04023-062 São Paulo, SP, Brazil; ∥ Department of Pharmacology, 58804 Federal University of São Paulo, CEP 04023-062 São Paulo, SP, Brazil; ⊥ Oswaldo Cruz Foundation, Leishmaniasis and Malaria Bioassay Platform, CEP 76812-245 Porto Velho, Rondônia, Brazil; # Research Center in Tropical Medicine of Rondônia, CEP 76812-329 Porto Velho, RO, Brazil; ∇ Museu Nacional, 28125 Universidade Federal do Rio de Janeiro, CEP 20940-040 Rio de Janeiro, RJ, Brazil; ○ Departamento de Química, Universidade Federal de São Carlos, CEP 13565-905 São Carlos, SP, Brazil

**Keywords:** Plasmodium falciparum, malaria, guanidine alkaloids, marine natural products

## Abstract

Malaria is a disease caused by pathogenic protozoa *Plasmodium* spp., with a significant global impact on human health. Increasing resistance of strains to drugs treating malaria highlights the urgent need for the discovery of new antimalarial candidates. Batzelladines are marine guanidine alkaloids that exhibit potent antiparasitic activity. Herein, results of the parasitological profiling assessment of batzelladines F and L are reported. Both compounds exhibited potent antiplasmodial activity, moderate cytotoxicity, and suitable selectivity indexes. Batzelladines F and L are fast-acting inhibitors, with a pronounced inhibitory activity against resistant strains and laboratory-adapted clinical isolates of . Batzelladines F and L also demonstrated *ex vivo* activity against clinical isolates of and , and batzelladine F showed *in vivo* antimalarial activity in a malaria model. The results reported constitute a robust rationale for the development of guanidine alkaloid derivatives as lead candidates for malaria treatment.

## Graphical abstract

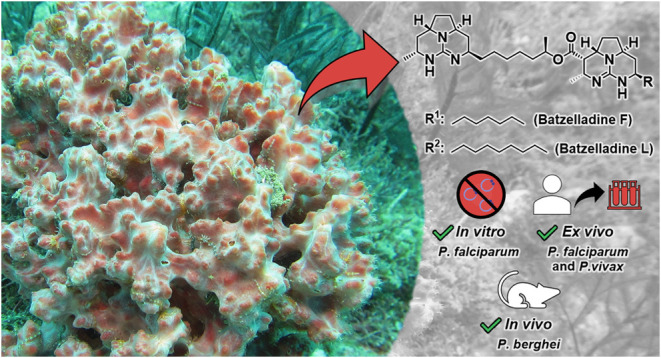

Malaria is an infectious disease caused by pathogenic protozoa belonging to the genus *Plasmodium spp.* Malaria has a significant global impact on human health, primarily due to its substantial burden of morbidity and mortality.^1,2^ In 2023, the World Health Organization (WHO) reported 263 million new cases of malaria and approximately 600 thousand deaths caused by the disease.^3^ Among the five species of *Plasmodium spp.* that infect humans, is responsible for the most severe and deadly cases of the disease, particularly in the African region.^2–4^ Quinine derivatives have been used since the 19th century for the treatment of malaria.^4^ Currently, the standard drugs employed for malaria treatment encompass diverse chemical entities including 4-aminoquinoline and artemisinin derivatives.^1,5^ These drugs largely fulfill the criteria for effective antimalarials, particularly in combined therapies.^6,7^ Artemisinin derivatives play a crucial role in the treatment of malaria, being used in artemisinin-based combination therapies (ACT) for uncomplicated and severe malaria caused by .^8^ Nevertheless, a significant challenge with current antimalarials is the widespread emergence of drug-resistant strains. Resistant strains of to artemisinin and its derivatives, as well as to other standard antimalarial drugs, have been reported and are increasingly spreading around the world.^9–12^ Consequentially, there is a very urgent need to discover new drug candidates for the treatment of malaria.

Natural products have been the main source of inspiration for the discovery of antimalarial drugs.^5^ Guanidine alkaloids have been isolated from different sources and are attractive natural products derivatives because of their pronounced biological activity.^13–23^ Batzelladines constitute a class of guanidine alkaloids isolated from marine sponges that display antiparasitic activity against *Leishmania* spp., and *Plasmodium* spp.^14–16,19^ The (poly)­cyclic structural framework of these alkaloids is of significant interest due to its varying degrees of carbon chain extension, number of guanidine moieties and the presence of monosubstituted guanidine chains (Figure 1).^13–19^ Moreover, the nitrogen-rich composition of natural guanidine derivatives make them well-suited candidates for inclusion in antiplasmodial screening programs.^24^ Several guanidine alkaloids, including batzelladines, have been assessed for their antiplasmodial activity,^14,15,19^ among which batzelladines F (**1**) and L (**2**) were reported as the most potent antiplasmodial agents.

**Figure 1:**
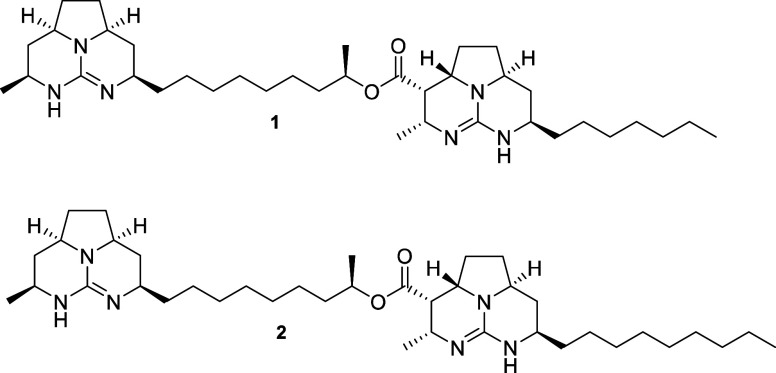
Chemical structures of batzelladines F (**1**) and L (**2**).

Herein we report the large-scale isolation of batzelladines F (**1**) and L (**2**) aiming to investigate the antiplasmodial profiling of these alkaloids, including detailed *in vitro*, *ex vivo*, and *in vivo* studies.

## Results

### Batzelladines L and F Showed Submicromolar Antiplasmodial Activities and Moderate Cytotoxic Activities

The marine guanidine alkaloids batzelladine F (**1**) and batzelladine L (**2**) were isolated in large amounts from the organic-soluble extract obtained from the sponge collected at the Brazilian coast. The MeOH extraction solvent was subjected to a series of liquid–liquid partitionings, providing an EtOAc extract which was subjected to size-exclusion chromatography. Selected fractions were purified by HPLC to yield batzelladines F (**1**, 40 mg) and L (**2**, 100 mg), identified by analysis of spectroscopic data and comparison with literature data.^19,22^ The purity of both alkaloids **1** and **2** were evaluated as >95% by ^1^H NMR analysis (Figures S5 and S15).

Batzelladines F (**1**) and L (**2**) were assayed *in vitro*, against (3D7 strain, chloroquine-sensitive) and against human hepatocellular carcinoma cells (HepG2 cell line), to evaluate their antiplasmodial and cytotoxic activities, respectively (Table 1).

**Table 1:** Evaluation of the *In Vitro* Antiplasmodial Activity (IC_50_), Cytotoxicity (CC_50_) and Selectivity Index Values of Batzelladines L and F

compound	IC_50_^3D7^ (μM) mean ± SD	CC_50_^HepG2^ (μM) mean ± SD	SI^a^
batzelladine F (**1**)	0.13 ± 0.01	10.6 ± 0.3	85
batzelladine L (**2**)	0.4 ± 0.1	14 ± 1	36

a SI = CC_50_/ IC_50_

Batzelladine L (**2**) displayed inhibitory activity against in the submicromolar range (IC_50_^3D7^ = 0.4 μM). This finding agrees with the previously observed inhibitory activity of batzelladine L against (IC_50_ = 0.3 μM, FcB1 strain, resistant to chloroquine).^15^ Moreover, compound **2** displayed moderate cytotoxic activity against HepG2 cells (CC_50_^HepG2^ = 14 μM), with a resulting selectivity index (SI) of 36. Compounds with SI values above 10 are considered promising for the discovery of new candidates for antimalarial drugs.^25^ Batzelladine F (**1**) (IC_50_ = 0.13 μM) exhibited a 3-fold increased inhibitory potency when compared to batzelladine L, but a similar cytotoxic effect on HepG2 cells (CC_50_ = 10.6 μM), thereby determining a 2-fold increased selectivity index (SI = 85) compared to batzelladine L (**2**).

### Batzelladines L and F Are Fast-Acting Inhibitors of 

Given the potent and selective antiplasmodial activity of batzelladines F (**1**) and L (**2**), a series of assays were conducted to characterize the parasitological profile of these compounds. We first evaluated if **1** and **2** were fast- or slow-acting inhibitors by assessing the inhibitory activity at three times of inhibitor exposure (24, 48, and 72 h). In this assay, the IC_50_ values of both compounds and two controls (artesunate and pyrimethamine, fast- and slow-acting inhibitors, respectively) were obtained at each exposure time and then compared to each other. Additionally, we implemented an extended assay protocol in which test samples were assayed using initial treatment periods of 24, 48, and 72 h, followed by regrowth periods of 6, 5, and 4 days, respectively, after inhibitor removal. This experimental design enabled us to determine whether a compound acts specifically within the first 24 h or if its inhibitory activity persists beyond this time frame.^26^ Fast-acting inhibitors maintain consistent inhibitory activities across all three exposure times, including the extended days after inhibitor withdrawal, whereas slow-acting inhibitors exhibit decreased IC_50_ values in the time frames of greater exposure to the inhibitor (e.g., 48 and 72 h). Batzelladines F and L showed comparable IC_50_ values in the three exposure times (Figure 2A). By contrast, pyrimethamine exhibited the expected behavior for a slow-acting inhibitor, with increased inhibitory potency observed at 48 and 72 h related to 24 h (Figure 2A). In the extended protocol, IC_50_ measurements taken 4 days after inhibitor pressure (IP) revealed comparable potency values across the ring, trophozoite, and schizont stages. The results indicated that the first 24 h of IP was sufficient to impair parasite development, regardless of the initial stage of incubation. Notably, these findings were consistent with the fast-acting positive control, artesunate (Figure 2B). These findings indicated that both batzelladines **1** and **2** are fast-acting inhibitors. In parallel, we assessed the morphological development of the parasite to confirm the speed of action of alkaloids **1** and **2**. In the negative control group (without inhibitor pressure), the parasites exhibited the expected development life cycle, including the ring stages at 0 h, trophozoites at 24 h, and rings and trophozoite forms again at 48 and 72 h, respectively (Figure 2C). For the fast-acting inhibitor artesunate, parasite death became evident in the first 24 h, characterized by the presence of pyknotic nuclei, which are not part of the normal development of the parasite and indicative of cellular death. As for the slow-acting inhibitor pyrimethamine, a 24 h exposure to the drug was not enough to induce the parasite death, since at this time the parasites developed normally, comparable to the negative control. Both batzelladines (Figure 2B,C) demonstrated similar behaviors to artesunate. These findings agreed with the assessed IC_50_ values (Figure 2A,B) and confirmed that batzelladines F and L are fast-acting inhibitors.

**Figure 2:**
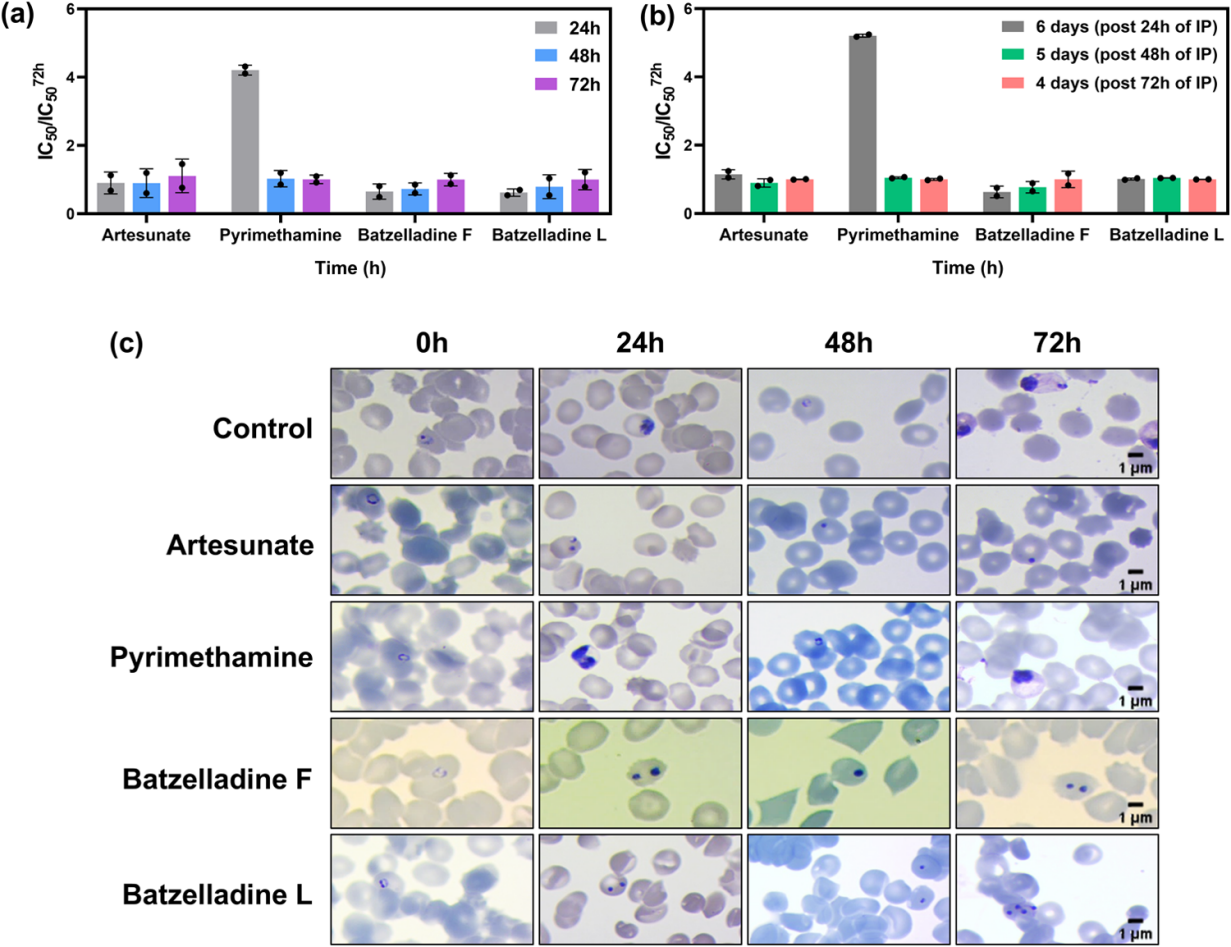
Speed of action evaluation of batzelladines F and L. (A) Inhibitory activities of the natural compounds in each time of exposure were determined and normalized in relation to the IC_50_ value assessed at 72 h. (B) Inhibitory activities of batzelladines F and L in each time of exposure after 4, 5, and 6 days of regrowth were determined and normalized in relation to the IC_50_ value assessed at day six. Day 6 corresponds to the plate after 24 h of inhibitor pressure (IP), Day 5 corresponds to the plate after 48 h of IP, and Day 4 corresponds to the plate after 72 h of IP. (C) Morphological evaluation of parasite in the absence (control) and presence of batzelladines L and F, artesunate (fast-acting inhibitor), and pyrimethamine (slow-acting inhibitor) controls (scale bar = 1 μm).

### Batzelladine L Acts in the Ring and Trophozoite Stages of the Parasite

To gain deeper insights into the parasitological profile of batzelladines, we assessed the stage specificity of inhibition within the intraerythrocytic cycle. Briefly, the assay consisted in evaluating the IC_50_ values of an inhibitor (batzelladine F or L) against distinct stages of the parasite, namely the early ring, late ring, early trophozoite, late trophozoite, and schizont stages. The obtained IC_50_ values for each stage were then compared to the IC_50_ values assessed at 72 h. Batzelladine L (**2**) was selected as a representative compound for this assay because it was isolated in much higher amounts.

Batzelladine L demonstrated IC_50_ ratio values comparable to those observed for chloroquine (positive control). Consequently, batzelladine L displayed enhanced inhibitory activities on both the ring (early and late) and trophozoite (early and late) stages (Figure 3B,C). It is noteworthy that batzelladine L exhibited inhibitory activity on the early ring stage (IC_50_ = 0.4 ± 0.1 μM), closely resembling that observed in the standard 72-h assay (IC_50_ = 0.21 ± 0.01 μM). These findings not only reinforce the results of the speed of action assay but also contribute to a more comprehensive understanding of batzelladine mode of action.

**Figure 3:**
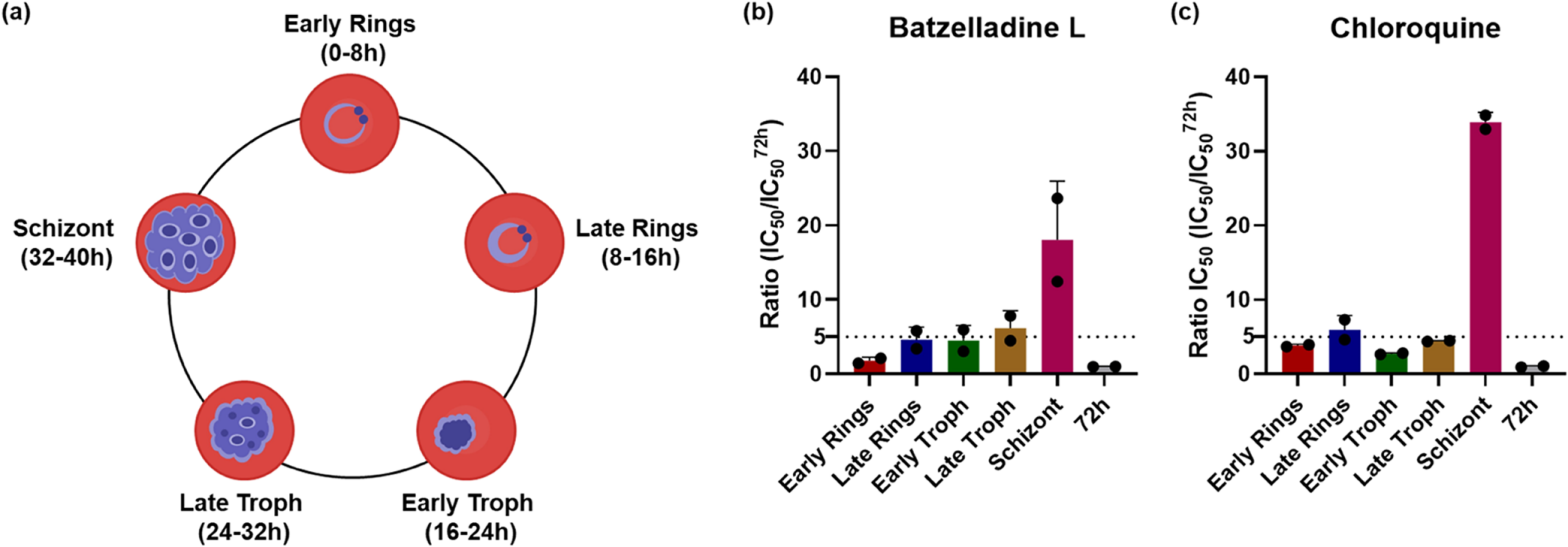
(A) Stage-specificity assessment representation of intraerythrocytic development evaluated in the assay. (B) IC_50_ ratio values of batzelladine L (**2**) in each time of exposure in relation to the 72 h IC_50_ value. (C) IC_50_ ratio values of chloroquine (control) in each time of exposure in relation to the 72 h IC_50_ value.

### Batzelladines Show an Antagonistic Combination with Artesunate and Chloroquine

To verify the potential use of batzelladines F (**1**) and L (**2**) as partners in artemisinin-based combination therapies (ACTs), we evaluated their combination with artesunate. Both batzelladines were combined with artesunate at eight fixed ratios (1:0, 6:1, 5:2, 4:3, 3:4, 2:5, 1:6, and 0:1). In addition, batzelladine L (**2**) was combined with chloroquine. The additivity isobole was determined using the Hand model.^27,28^ Statistical analysis was employed to assess the combination effects. The absence of a statistical difference between the assessed values and the additivity isobole indicated an additive inhibitor combination. Conversely, statistically different curves suggested a synergistic (values below the additivity curve) or antagonistic (values above the additivity curve) inhibitor combination. The combination of batzelladines F (**1**) and L (**2**) with artesunate exhibited a pronounced antagonistic profile (Figure 4A,C, respectively). The experimental data points (red dots and red region) were notably above the additivity curve (black line and gray region). Statistical analysis confirmed a significant difference between the experimental data and the additivity isoboles (*p*-values of 0.027 and 0.0005, Figure 4B,D, respectively), providing statistical support for the observed antagonistic combination profiles. A comparable antagonistic combination profile was observed for batzelladine L (**2**) with chloroquine (*p*-value: 0.0018, Figure S3). Consequently, batzelladines exhibited an antagonistic combination profile when combined with artesunate and chloroquine.

**Figure 4:**
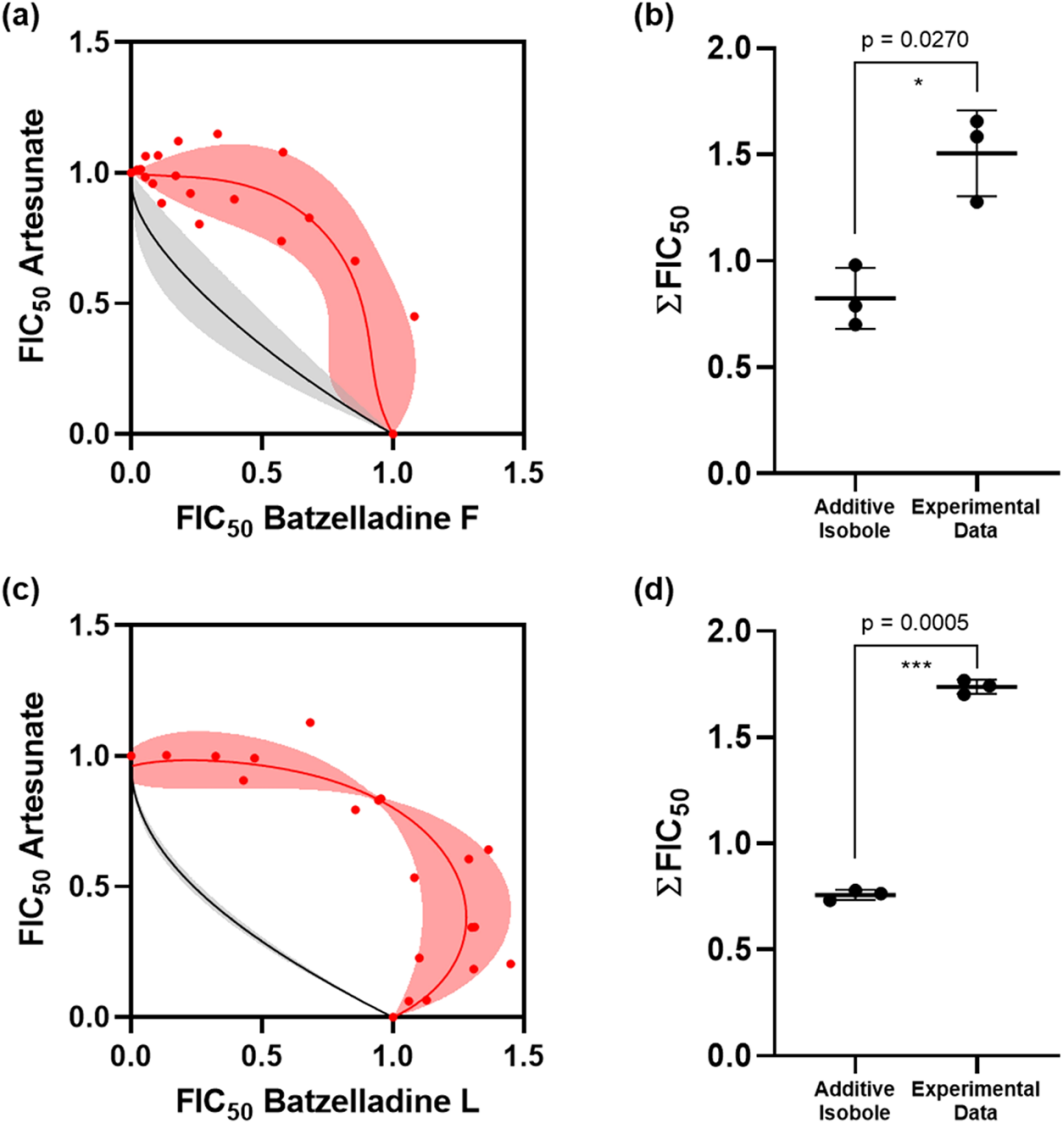
Assessment of the combination of batzelladines L and F with artesunate. The red region and red dots represent the experimental data, while the black line and gray region indicate the additivity curve. Isobolograms of batzelladines F (A) and L (C) combined with artesunate. Statistical analysis was performed for the combination of batzelladines F (B) and L (D) with artesunate. These represent the ∑F IC_50_ values of three independent experiments (*p*-value <0.05 shows statistical difference between the experimental data and the additivity isobole).

### Batzelladines F and L Are Potent Inhibitors of Resistant Strains

The subsequent phase in exploring the antiparasitic properties of batzelladines F and L involved evaluating their inhibitory activity against a representative panel of resistant strains. This assessment aimed to ascertain whether the batzelladines exhibit cross-resistance with standard antimalarials. resistant strains in the panel included: Dd2 (resistant to chloroquine, sulfadoxine, pyrimethamine, mefloquine, and cycloguanil), K1 (resistant to chloroquine, sulfadoxine, pyrimethamine, and cycloguanil), Dd2R_DSM265 (a Dd2-derived strain resistant to DSM265, a PfDHODH inhibitor), and 3D7R_MMV848 (a 3D7-derived strain resistant to MMV692848, a PfPI4K inhibitor). The IC_50_ values for each resistant strain were determined, and a resistance index (RI) was calculated as the ratio of IC_50_^Resistant strain^ to IC_50_^3D7^ (Table 2). RI values greater than five indicate cross-resistance.^29^ Batzelladines F and L exhibited comparable IC_50_ values for both sensitive and resistant strains, resulting in RI values less than five (Figure 5), indicating that neither compound demonstrated cross-resistance with the standard antimalarials used as controls for each resistant strain.

**Table 2:** Evaluation of Batzelladines F (**1**) and L (**2**) *In Vitro* Antiplasmodial Activity (IC_50_) for Each Resistant Strain and Their Respective Resistance Index (RI)

	IC_50_ (nM) (IC_50_ row must include the last two columns (3D7R_MMV265 and RI)								
compound	3D7	Dd2	RI	K1	RI	Dd2^R^_DSM265	RI	3D7^R^_MMV848	RI
batzelladine F	110 ± 20	130 ± 80	1.5	108 ± 5	1	160 ± 60	1.4	130 ± 50	1.2
batzelladine L	170 ± 50	270 ± 90	1.5	350 ± 50	2	350 ± 50	2	230 ± 60	1.4
artesunate	11 ± 4	10 ± 5	0.9	5.3 ± 0.8	0.5	12 ± 3	1.2	13 ± 3	0.8
pyrimethamine	37 ± 6	>10,000	>859	>10,000	>392				
DSM265	3.4 ± 0.8					19 ± 5	5.5		
MMV692848	84 ± 8							>1000	>20

**Figure 5:**
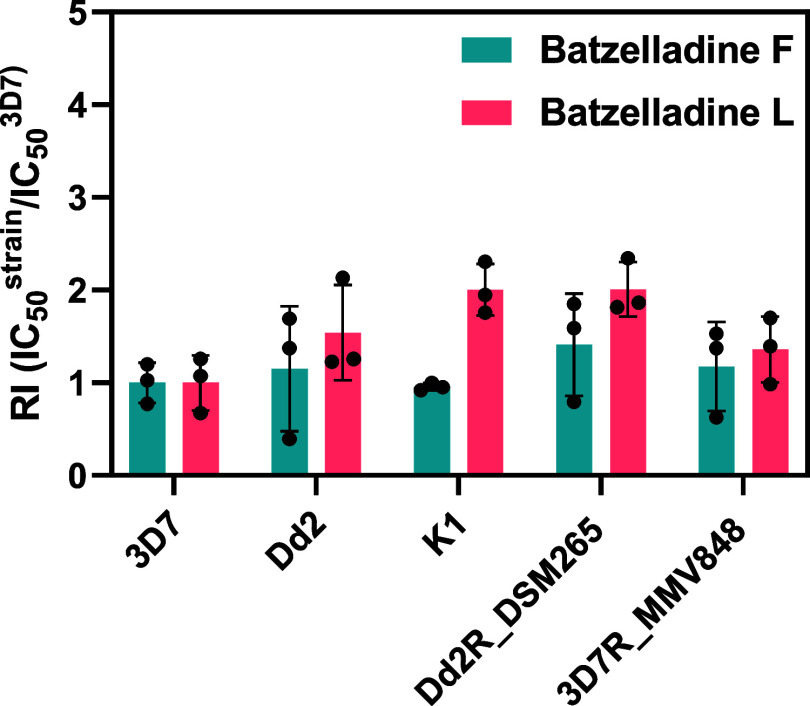
Resistance index of batzelladines F and L against a panel of resistant strains (Dd2, K1, and Dd2^R^_DSM265 and 3D7^R^_MMV848) in relation to the sensitive 3D7 strain.

### Batzelladines F and L Showed Inhibitory Activity Against Laboratory-Adapted Strains of from Brazilian Field Isolates

The subsequent phase of our investigation involved assessing the inhibitory activity of batzelladines against field isolates adapted *in vitro*. These isolates were originally sourced from blood samples collected from patients infected with in the region of Porto Velho, Rondônia (malaria-endemic area in Brazil) and adapted to *in vitro* laboratory culture. This assay enables the evaluation of the IC_50_ values of batzelladines F and L in the adapted strains and facilitates a comparison with the IC_50_ value in the sensitive strain (3D7). Furthermore, this offers insights into the activity of these compounds in strains that were actively circulating in the field. The laboratory-adapted strains employed in the assay included parasites collected from three different patients namely: BRPVH-39, BRPVH-41 and BRPVH-45. With the determination of the IC_50_ value for each laboratory-adapted strain, a ratio (R) value was calculated by the ratio of IC_50_^Adapted strain^ to IC_50_^3D7^ (Table 3).

**Table 3:** Evaluation of Batzelladines F (**1**) and L (**2**) *In Vitro* Antiplasmodial Activity (IC_50_) on Field Isolates Adapted *In Vitro* and Their Ratio (R) Relative to the Sensitive Strain (3D7)

	IC_50_ (nM)						
compound	3D7	BRPVH-39	R^a^	BRPVH-41	R^a^	BRPVH-45	R^a^
batzelladine F	110 ± 20	80 ± 20	0.7	80 ± 30	0.7	90 ± 50	0.8
batzelladine L	170 ± 50	440 ± 50	2.5	340 ± 70	2.0	430 ± 100	2.5

a R = IC_50_^Adapted strain^/ IC_50_^3D7^

Batzelladines F and L showed comparable IC_50_ values for the sensitive and adapted strains (Figure 6) and, consequently, the *R* values were less than five. This indicates that both compounds are active against laboratory-adapted strains and could be effective in the field.

**Figure 6:**
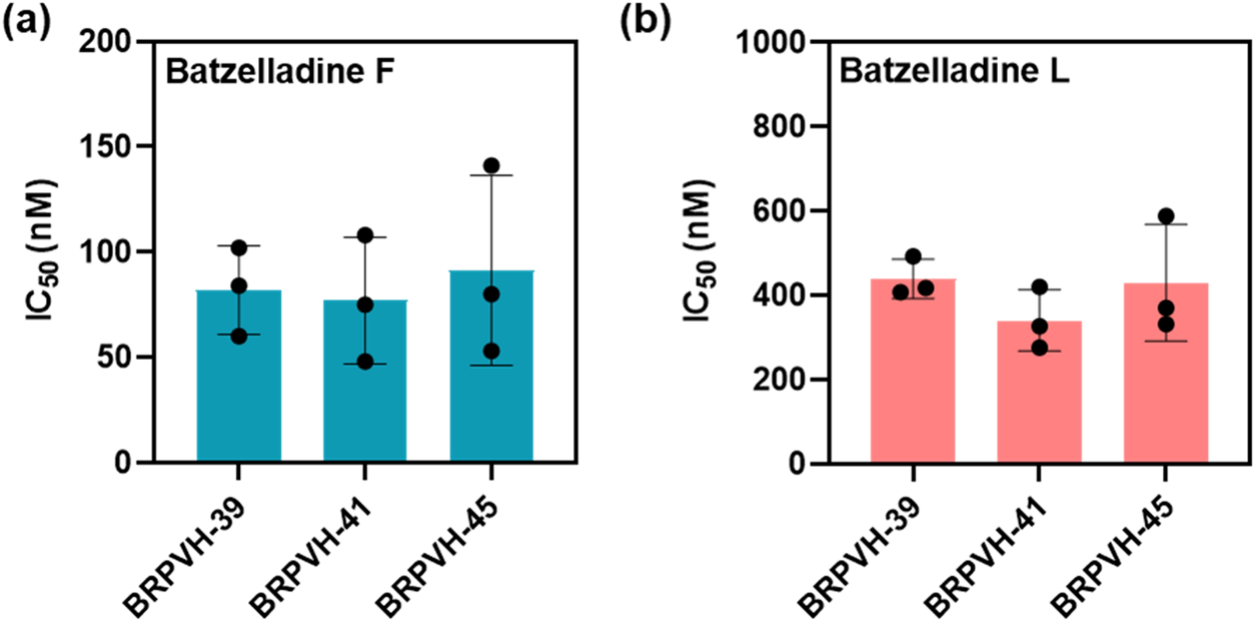
Inhibitory activity for three laboratory-adapted strains (BRPVH-39, BRPVH-41 and BRPVH-45). (A) Batzelladine F IC_50_ values. (B) Batzelladine L IC_50_ values. The data represent the IC_50_ values obtained from three independent experiments.

### Batzelladine L Inhibits and Field Isolates

Given the notable potency of batzelladines against laboratory-adapted strains, we conducted an *ex vivo* schizont maturation assay (SMT) to validate and confirm the inhibitory activity of batzelladines against circulating field isolates. This assay is particularly significant as it assesses the antiplasmodial activity of compounds against presently circulating strains of the parasite.^30^ Moreover, the assay offers the opportunity to test the compounds against another clinically relevant *Plasmodium* species, such as . Thus, clinical isolates of and collected from patients in the Porto Velho region of Rondônia in 2022 were used. Batzelladines F and L were active against and isolates (Table 4, Figure 7). Notably, batzelladine F showed increased potency against isolates (IC_50_ value of 40 nM with a range of 19–641 nM in the individual isolates). These findings indicate that batzelladines F and L exhibited activity against currently circulating parasite strains.

**Table 4:** Evaluation of Batzelladines F and L *Ex Vivo* Antiplasmodial Activity (IC_50_) Against Circulating Strains of and in the Schizont Maturation Assay (SMT)

	*Ex vivo* IC_50_ (median [interval], nM)	
compound		
batzelladine F	670 [150–3570] (*n* = 6)	40 [19–641] (*n* = 7)
batzelladine L	1222 [618–1300] (*n* = 5)	632 [153–1295] (*n* = 9)

**Figure 7:**
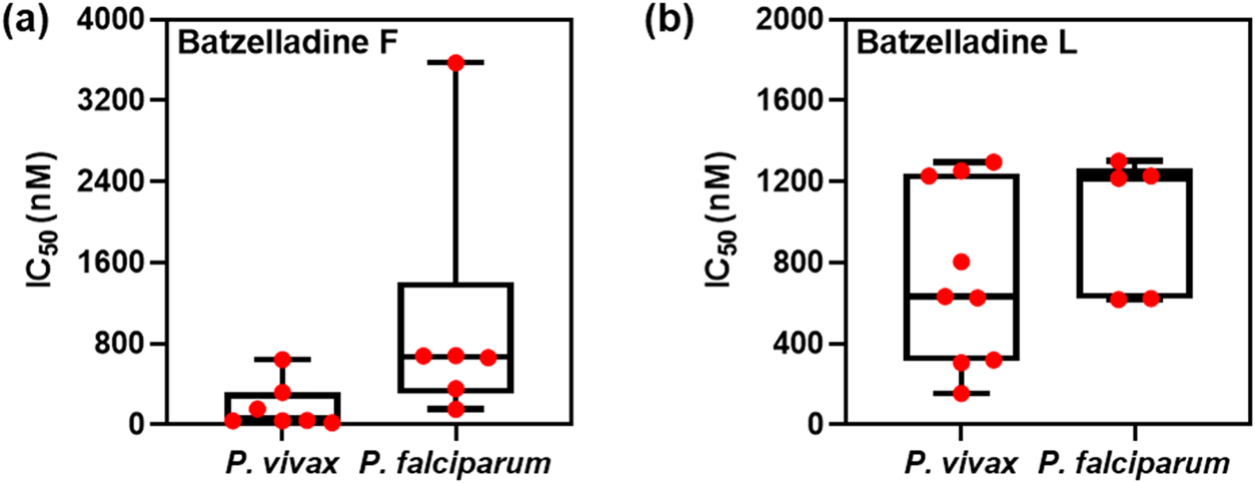
Distribution of IC_50_ values obtained in the *ex vivo* schizont maturation assay (SMT) against circulating field isolates for (A) Batzelladine F (, *n* = 6 *and* , *n* = 7) and (B) Batzelladine L (, *n* = 5 *and* , *n* = 9).

### Batzelladine F is Active Against in the *In Vivo* Model

The promising activity demonstrated by batzelladines F (**1**) and L (**2**) in the *in vitro* and *ex vivo* assays motivated us to assess the antimalarial activity in an *in vivo* model of the diseases using mice infected with (NK65 strain). Due to the limited quantity of batzelladine F available for testing, we use Peters’ suppressive test with minor adaptations. The reduction in percentage of parasitemia was calculated in relation to the untreated control group on days 5, 8, and 11 postinfection, after the administration of 50 mg/kg/day in the treated group (Table 5). Batzelladine F (**1**) was selected as a representative compound for this assay. Chloroquine was used as a positive control at a dose of 20 mg/kg. Batzelladine F (**1**) exhibited a notable 94% reduction in parasitemia on day 5 postinfection (Figure 8A), followed by sustained reductions of 60 and 77% on days 8 and 11 postinfection, respectively. Additionally, analysis of survival rates in the treated and untreated groups revealed that the batzelladine F-treated group displayed a survival rate significantly greater than that of the untreated control group (Figure 8B). The survival rate in the batzelladine F-treated group was comparable to that observed in the chloroquine-treated group. These results indicated that treatment with alkaloid **1** was well-tolerated and demonstrated pronounced antimalarial activity, providing protection in a mouse malaria model.

**Table 5:** *In Vivo* Antimalarial Activity of Batzelladine F (**1**) and Chloroquine in Mice Infected with

		% parasitemia			% parasitemia reduction		
compound	dosage (mg/kg)	5^th^	8^th^	11^th^	5^th^	8^th^	11^th^
batzelladine F (**1**)	50	0.1 ± 0.1	1.6 ± 0.6	1.4 ± 0.6	94	60	77
chloroquine	20	0	0	0	100	100	100
control		3 ± 2	5 ± 2	11 ± 3			

**Figure 8:**
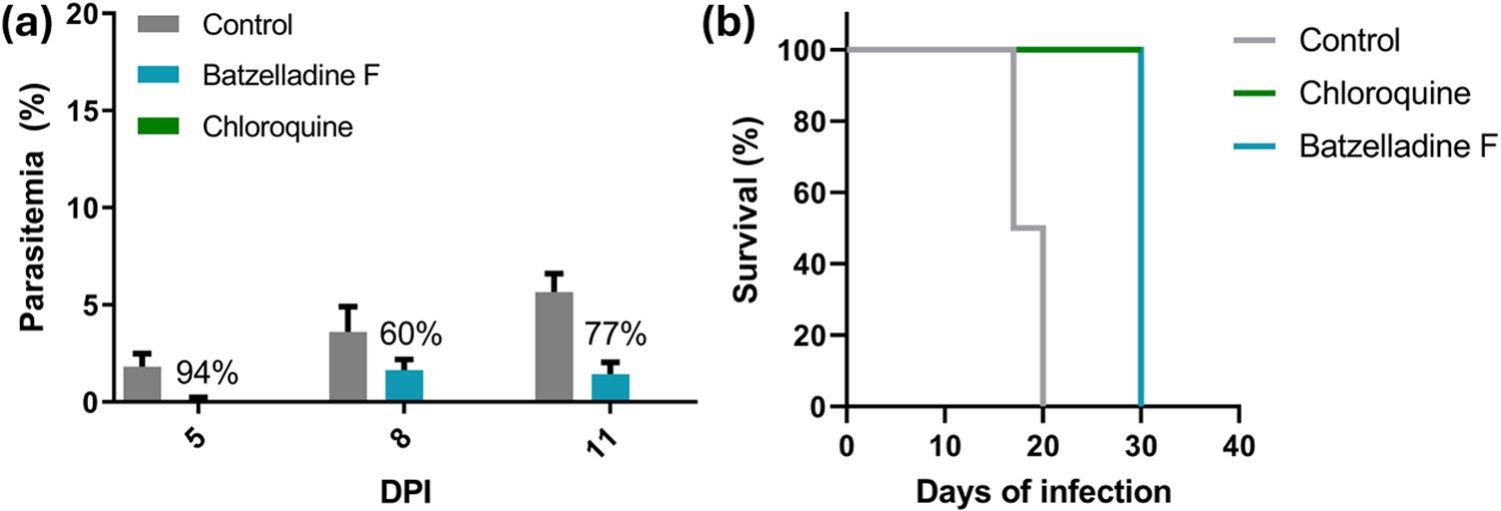
*In vivo* antimalarial activity assessment of batzelladine F (**1**). (A) Percentage of reduction in parasitemia of batzelladine F (**1**) in relation to the control. (B) Survival (%) of mice infected with (strain NK65) treated with Batzelladine F (**1**) and chloroquine, evaluated during the experiment (30 days). DPI = Days postinfection.

## Discussion

Batzelladine L (**2**) is a tricyclic guanidine alkaloid with a reported antiplasmodial activity against FcB1 strain (IC_50_ = 0.3 μM).^15,31^ However, the antiplasmodial activity of batzelladine F (**1**) against has not yet been investigated.^13^ The structural distinction between these two compounds is two additional methylene groups in batzelladine L when compared to batzelladine F (Figure 1). Thus, we assume that batzelladine F (**1**) is less lipophilic than batzelladine L (**2**), and we considered relevant to evaluate this difference for the antiplasmodial activity profile of these two alkaloids. Indeed, while batzelladine L showed antiplasmodial activity against the chloroquine-sensitive 3D7 strain (IC_50_^3D7^ = 0.4 μM) very similar to previously reported data,^13^ batzelladine F proved to be 4-fold more potent against (IC_50_^3D7^ = 0.13 μM) than batzelladine L under the same assay conditions. Moreover, batzelladines L and F exhibited moderate cytotoxic activity toward HepG2 cells (CC_50_s of 14 and 10.6 μM, respectively) and suitable selectivity indexes (SI values of 36 and 85, respectively), indicating potential selective antiplasmodial activity, promising for additional investigations.

New antimalarial candidates that act as fast-acting inhibitors are preferred.^6^ Batzelladines F and L showed a comparable profile to the fast-acting control (artesunate) in the extended speed of action assay. The assessed IC_50_ values for the first 24 h of exposure to these alkaloids were comparable to the inhibitory activities at 72 h. Additionally, the parasites’ morphological analysis in this assay provided further support to the fast-acting profile of batzelladines F and L. Exposure of parasites to the batzelladines during 24 h, followed by a washing out procedure, was enough to induce the death of the parasites with no evidence of recovery.

In addition to the preference for fast-acting inhibitors, there is a significant interest for compounds that exhibit enhanced inhibitory activity on the early stages of the intraerythrocytic parasite development.^6^ Understanding the stage specificity of a *Plasmodium ssp.* inhibitor can also provide valuable insights into the drug candidate MoA.^32^

Therapies that combine different drugs are considered an advantageous strategy for the treatment of various diseases, including malaria.^33^ In the combination assays, both batzelladines L and F displayed an antagonistic combination profile with artesunate, and batzelladine L showed an antagonistic combination profile with chloroquine, as evidenced by the curves of experimental data notably above the additivity isobolograms. Despite the antagonistic combination profiles, batzelladines F and L hold promise for further combination analysis.

Assessing the efficacy of inhibitors against resistant strains is also important due to the ongoing challenge of drug resistance in malaria treatment.^9–12^ This evaluation helps us to determine their potential cross-resistance with standard antimalarials. Our results indicated that both batzelladines did not exhibit cross-resistance with the resistant strains tested (Dd2, K1, Dd2^R^_DSM265, and 3D7^R^_MMV848), a positive outcome in the context of combating drug-resistant malaria. Additionally, our results suggest that batzelladines L and F may show a distinct mode of action when compared with standard antimalarials.

Evaluating the antiplasmodial activity of candidate compounds against clinical isolates is relevant as well, since this assay offers valuable insights into the validation of the inhibitory activity of a particular compound against parasite strains actively circulating in the field.^6^ Two assays were conducted to assess the antiplasmodial activity of batzelladines L and F against clinical isolates. The first approach involved evaluating the inhibitory potency of the natural compounds against laboratory-adapted strains collected from field isolates. Batzelladines L and F showed comparable IC_50_ values for the sensitive and laboratory-adapted strains (Table 3), which could be indicative that the compounds may be effective in the field. In order to address this hypothesis, we conducted a second experiment to verify the *ex vivo* inhibitory activity of both compounds through the schizont maturation assay. This assay provided an opportunity to test the representative compound against , which is present in the American continent, East Africa, and Southeast Asia.^3^ The IC_50_ values for batzelladines F and L obtained against and isolates were consistent with the values observed in the *in vitro* experiment against the 3D7 strain and the laboratory-adapted strains. Our findings are of significant interest as they not only support the antiplasmodial activity of batzelladines against actively circulating strains but also demonstrate the alkaloid activity against another species of *Plasmodium spp*.

Our detailed analysis of batzelladines parasitological profile included the evaluation of the *in vivo* antimalarial activity of batzelladine F using a model.^34^ Batzelladine F reduced the parasitemia by 94% on day 5 postinfection. This value is considerably greater than the minimum threshold used for classifying a compound as active in an *in vivo* experiment (>30%).^35^ Moreover, the group treated with batzelladine F exhibited a pronounced survival rate compared to the untreated control group, meaning that treatment with batzelladine F protected the animals from *Plasmodium* infection.

In an attempt to rationalize the potential mode(s) of action for the batzelladines based on their parasitological profile, we have raised some hypotheses. The first involves the modulation of redox oxidative stress pathways. Our prior studies have shown that batzelladines can induce the generation of reactive oxygen species (ROS), which is their MoA for killing *Leishmania* parasites.^16^ Despite their distinct protozoan indoparasitism, *Leishmania* and *Plasmodium* share similarities in their redox and antioxidant systems.^36^ Therefore, it is reasonable to consider that batzelladines F and L may exert their biological activity against through oxidative stress. Compounds known to induce excessive oxidative stress, resulting in parasite death, are fast-acting agents, exhibit potent efficacy against ring-stage parasites, and do not show cross-resistance.^37–39^ On the other hand, unlike our compounds, these agents are expected to synergize with artemisinin-based drugs.

Another hypothesis we considered involves the batzelladines inhibition of the nucleoside transporter 1 of (*Pf*ENT1).^40,41^ Given that *Plasmodium* parasites are purine auxotrophs, the proteins involved in the purine salvage pathways are vital for parasite growth.^42,43^ Consequently, inhibitors of *Pf*ENT1 effectively eliminate parasites through purine starvation, proving to be fast-acting compounds as the parasites fail to advance beyond the ring stage.^44–46^ Furthermore, no cross-resistance was observed for inhibitors targeting *Pf*ENT1 in Dd2, HB3, 7G8 strains, and an artemisinin-resistant strain (ART^R^).^44,45^ Some of these *Pf*ENT1 inhibitors were also active against ENT1 from (*Pv*ENT1), suggesting that they could inhibit other *Plasmodium* species that infect humans. Compounds that inhibit *Pf*ENT1 are active against *Pb*ENT1 and have shown growth inhibition properties on parasites in *ex vivo* cultures.^46^ Based on these data, which are in agreement with the antiplasmodial properties of the batzelladines, we modeled the binding mode of batzelladines F (**1**) and L (**2**) to *Pf*ENT1 through molecular docking and molecular dynamics (MD) simulations.

Inhibitors of *Pf*ENT1 are characterized by having a hydrogen bond acceptor to interact with the amide side chain of Gln135.^45,47^ Also, a CH-π interaction with the aromatic side chain of Trp53 has been shown to be essential for the inhibitory activity.^45,47^

The modeled binding mode of batzelladines F and L indicated that the ester group in the chemical structures is in close contact with Gln135, and the alkyl chain presents favorable van der Waals interactions with Trp53 (Figure 9). Furthermore, the alkyl substituent interacts with the target through hydrophobic contacts, which could favorably contribute to the binding of the compounds to *Pf*ENT1. The protonated tricyclic guanidine moiety was involved in polar contact with the side chain hydroxyl group of Ser621.

**Figure 9:**
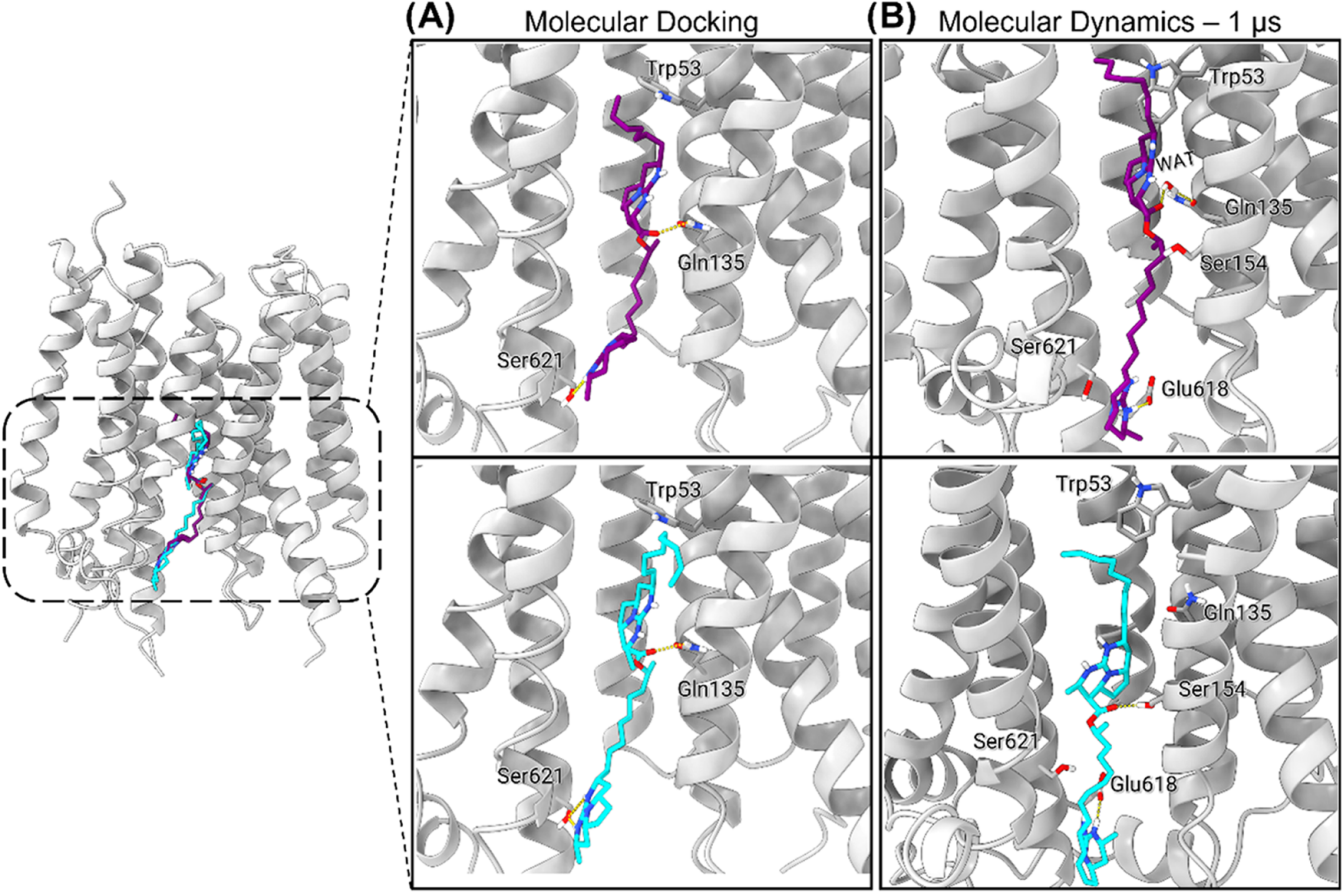
Modeled binding mode of batzelladines F (purple) and L (cyan) to *Pf*ENT1. (A) Putative binding mode generated by molecular docking. (B) Representative binding mode after 1 μs of MD simulations for each complex. Hydrogen bonds are represented as yellow dashed lines. Helix TM8 of *Pf*ENT1 and the membrane bilayer were omitted for clarity in the insets.

We validated the proposed binding modes of batzelladines F (**1**) and L (**2**) to *Pf*ENT1 using a thorough molecular dynamics (MD) simulation protocol, conducted for 1 μs for each inhibitor. The batzelladine F system stabilized rapidly within 30 ns, whereas the batzelladine L system required approximately 200 ns to reach stability (Figures S26 and S27). Notably, the batzelladine L system exhibited greater protein fluctuations compared to batzelladine F, as indicated by the root-mean-square fluctuations (RMSF) of the *Pf*ENT1 α carbons (Figures S26c and S27c). The simulations revealed that batzelladine F forms stable interactions with *Pf*ENT1, in particular, maintaining a critical interaction with Gln135. This finding is consistent with its greater potency against the parasite in cellular assays compared to batzelladine L. Overall, the MD simulations provided valuable insights into the putative binding modes of batzelladines F and L to *Pf*ENT1, corroborating the docking results and supporting our hypothesis that *Pf*ENT1 is a potential target for batzelladines.

## Conclusions

Herein, we undertook an extensive examination of the parasitological profile of the marine guanidine alkaloids batzelladines F and L isolated from Brazilian specimens of the tropical western Atlantic marine sponge . Both batzelladines F and L demonstrated antiplasmodial activity in the submicromolar range, exhibited a low cytotoxic effect on HepG2 cells, and had a favorable selectivity index. Both compounds were fast-acting inhibitors with batzelladine L primarily targeting the ring and trophozoite stages, especially the early rings. Batzelladines showed antagonistic combination profiles with artesunate and chloroquine, indicating that they may not be suitable partners in combination therapy. Both batzelladines were also effective against a representative panel of resistant strains and laboratory-adapted clinical isolates, demonstrated *ex vivo* inhibitory activity on clinical isolates of and and batzelladine F displayed a high *in vivo* activity against ; however, it will be important to evaluate its in vivo activity using a curative protocol. Based on the parasitological profile data of batzelladines F and L and molecular modeling analyses, we propose that batzelladines F and L can modulate the redox pathway or bind to *Pf*ENT1, a key transporter in the purine salvage pathway. From a biological perspective, our findings indicate that batzelladine derivatives are potentially promising hit candidates for nature-inspired antimalarial drug discovery programs.

## Methods

### General Experimental Procedures

Structural characterization methods and analytical HPLC and UPLC details are described in the Supporting Information file.

### Maintenance of *In Vitro* Culture

Continuous *in vitro* cultures of (strains 3D7, Dd2, K1, Dd2^R^_DSM265, and 3D7^R^_MMV848) were kept using an adaptation of the method described by Trager and Jansen.^48^ The parasites were cultivated in a humidified incubator at 37 °C, at low-oxygen atmosphere (5% O_2_, 5% CO_2_, 90% N_2_) in RPMI-1640 medium with 25 mM NaHCO_3_, 25 mM HEPES (pH 7.4), 11 mM d-glucose, 3.67 mM hypoxanthine, and 25 μg/mL gentamicin, supplemented with 0.5% (m/v) AlbuMAX II. The culture medium was changed daily, and the parasitemias were maintained below 10% with 2.5% hematocrit. All compounds were purchased from Sigma-Aldrich (Cotia, Brazil).

### SYBR Green I Growth Inhibition Assay Against Asexual Forms

Initially, the antiplasmodial activities of batzelladines L and F were evaluated against blood parasite 3D7 (chloroquine-sensitive). Compounds were diluted to a stock concentration of 20 mM in 100% DMSO before the experiments and maintained at −20 °C. The parasites were synchronized through sterile 5% (m/v) D-sorbitol treatment over 10 min at 37 °C for the enrichment of ring-stage parasites.^49^ Centrifugation 600*g* over 5 min was used to pellet the cultures. The parasitemia was determined by the microscope analysis of thin blood smears stained with Giemsa 10% (v/v) after fixation with methanol. The initial parasitemia was calculated for 1000 red blood cells (RBCs), and cultures were diluted to 0.5% parasitemia and 2% hematocrit by the addition of the appropriate volumes of blood and medium. Parasite aliquots of 180 μL were distributed into 96-well plates previously prepared with 20 μL aliquots of a 10-fold concentrated compound, and the range of concentrations tested was 10–0.097 μM for batzelladine L and 1–0.0097 μM for batzelladine F (Figure S1). Negative and positive control wells corresponding to nonparasitized erythrocytes and parasite cultures in the absence of compounds were set in parallel. The DMSO concentration was maintained below 0.05% (v/v). The plates were incubated for 72 h at 37 °C in a humidified incubator with a gas mixture of 90% N_2_, 5% O_2_, and 5% CO_2_. Each test was performed in duplicate, and the results were compared to the control cultures. After incubation, the culture medium was removed, and the cells were resuspended in 100 μL PBS buffer (116 mM NaCl, 10 mM Na_2_HPO_4_, 3 mM KH_2_PO_4_) and lysed with 100 μL lysis buffer (20 mM Tris base, 5 mM EDTA, 0.0008% (v/v) Triton X-100, 0.008% (m/v) saponin, pH 8.0) containing 0.002% (v/v) SYBR Green I.^50^ The plates were incubated for an additional 30 min, after which the fluorescence of the plate was measured using a SpectraMAX Gemini EM plate reader (Molecular Devices Corp., Sunnyvale, CA) (λ_ex_ = 485 nm, λ_em_ = 535 nm). Fluorescence intensity was analyzed in terms of parasite viability as compared to controls using the software GraphPad Prism version 8.0.1 (GraphPad Software, San Diego, CA). Concentration–response curves were built, and half-maximal inhibitory concentration (IC_50_^Pf^) values were determined for each compound using nonlinear regression analysis.

### Cultivation of Human Hepatocellular Carcinoma Cells (HepG2 Cell Line)

The cytotoxic effects of batzelladines L and F were evaluated against the human hepatocellular carcinoma cell line (HepG2). The culture of HepG2 cells was kept in a flask in a humidified atmosphere of 5% CO_2_ at 37 °C, and every 2 days, the supplemented medium was changed. The culture medium used was RPMI-1640 supplemented with 25 mM HEPES (pH 7.4), 24 mM sodium bicarbonate, 11 mM d-glucose, 40 μg/mL penicillin–streptomycin, and 10% (v/v) bovine fetal serum. Every 3–4 days, treatment with a 0.25% trypsin solution was used to release cells from the flask walls, and a 1:4 proportion of the cells were maintained in culture.

### Resazurin Assay for Cytotoxicity Evaluation

The HepG2 cells were trypsinized, counted, and distributed in a 96-well plate, in a proportion of 30,000 cells per well (180 μL). The plate was incubated at 37 °C and 5% CO_2_ for 24 h to allow cell adhesion. After 24 h, 20 μL of serial dilutions of the compounds tested were added to the plate, with a range of concentrations tested from 50 to 0.78 μM. The plate was incubated for 72 h at 37 °C and 5% CO_2_. Cells without any treatment were maintained as positive controls, and wells containing only medium were used as negative controls. After incubation, a microscope was used to determine the highest compound concentration to be considered in the treatment results (highest concentration without precipitation). The cytotoxicity of the compounds was evaluated by adding 40 μL of resazurin (0.15 mg/mL) to each well, and the plates were kept for 4 h at 37 °C and 5% CO_2_. The fluorescence of the plate was measured using a SpectraMAX Gemini EM plate reader (Molecular Devices Corp., Sunnyvale, CA) (excitation wavelength of 560 nm, emission wavelength of 590 nm). Fluorescence intensity was analyzed in terms of cell viability as compared to controls using the software GraphPad Prism version 8.0.1 (GraphPad Software, San Diego, CA). Concentration–response curves were generated, and half-maximal inhibitory concentration (CC_50_) values were determined for each compound using nonlinear regression analysis (Figure S1). The selectivity index (SI) was calculated by the ratio of CC_50_ to IC_50_. Of note, compounds with an SI over 10 are generally considered selective.

### Speed of Action Assay

To determine whether batzelladines L and F were fast- or slow-acting inhibitors, a protocol adapted from Terkuile and collaborators (1993)^51^ was performed. samples at the ring stage were used to prepare a 0.5% parasitemia and 2% hematocrit mixture, which was then distributed in three 96-well plates. A serial dilution of the compounds was added to each plate; the starting concentration for batzelladine L was 10 μM, for batzelladine F 1 μM, and for artesunate and pyrimethamine was 10 × IC_50_ (fast- and slow-acting controls, respectively). Each plate was treated with the compounds for a different time (24, 48, and 72 h). The first two plates were washed twice with fresh medium to remove the inhibitor, followed by incubation of 48 and 24 h, respectively. At the end of 72 h, all plates were evaluated using the SYBR Green I assay to determine compound inhibitory activity at each time point. Figure S4 shows an assay scheme. After drug removal, the compounds are tested with initial treatments of 24, 48, and 72 h, followed by 6, 5, and 4 days of regrowth, respectively. At the end of 96 h, all plates were evaluated using the SYBR Green I assay to determine compound inhibitory activity at each time point. In parallel, the morphological development of the parasite under the compounds’ pressure was assessed by adding the compounds tested at the highest concentration evaluated for 24 h. After 24 h, this plate was washed twice with fresh medium to remove the inhibitor, and blood smears of each well were made and stained at time points 24, 48, and 72 h. A blood smear of a positive control, which corresponded to parasite cultures with no addition of inhibitor, was made at time points 0, 24, 48, and 72 h for the parasite’s control growth.

###  Stage-Specificity Inhibition Assay

To evaluate the batzelladine L activity on different stages of intraerythrocytic development, we performed a protocol adapted from Murithi et al.^32^ A sample was tightly synchronized at the ring stage using an MACS magnetic LS column, which was used to prepare a mixture with 0.5% parasitemia and 2% hematocrit. This mixture was then distributed in six 96-well plates. Five plates were used to assess the compound inhibitory activity during specific 8 h time intervals corresponding to different intraerythrocytic stages of the development: Plate A (0–8 h, early ring), Plate B (8–16 h, late ring), Plate C (16–24 h, early trophozoite), Plate D (24–32 h, late trophozoite), and Plate E (32–40 h, schizont). Plate A, corresponding to early rings, was incubated with batzelladines F and L from 0 to 8 h and after 8 h of exposure, iRBCs were washed twice with RPMI-1640 medium. Next, the alkaloids were added to plate B and incubated from 8 to 16 h postinfection, corresponding to late ring stage. The same protocol was performed for all five plates (A, B, C, D, and E) in each time point. Regardless of the parasite’s exposure time to the batzelladines, all plates were maintained at 37 °C in a humidified incubator with a gas mixture of 90% N_2_, 5% O_2_, and 5% CO_2_ for 60 h. At the end of 60 h, the parasite viability for all plates was assessed using the SYBR Green I assay to determine the alkaloids’ inhibitory activity at each developmental stage.^26^ An additional plate was used to evaluate the antiplasmodial potency of batzelladines in the standard 72 h assay, as previously described. Figure S5 shows the assay scheme. Fluorescence intensity was analyzed in terms of parasite viability as compared to controls, using the software GraphPad Prism version 8.0.1 (GraphPad Software, San Diego, CA). Concentration–response curves were generated, and half-maximal inhibitory concentration (IC_50_) values were determined for each time exposure by using nonlinear regression analysis.

### Combination Assay with Artesunate

This assay was adapted from the work done by Fivelman and collaborators (2004).^52^ The compounds and artesunate were diluted and combined in a 96-well plate in seven fixed-ratio combinations (1:0, 6:1, 5:2, 4:3, 3:4, 2:5, 1:6, and 0:1). Starting concentrations were 10 × IC_50_ for all compounds, and the experiment was performed with 0.5% parasitemia and 2% hematocrit. Serial dilutions of these combinations were prepared and incubated with the parasite, as described above, to determine the antiplasmodial activity against . The SYBR Green I test was applied to determine the IC_50_ value for each combination using the software GraphPad Prism version 8.0.1 (GraphPad Software, San Diego, CA). The additivity isobole was determined using the Hand model, as previously described.^27,28^ Fractional inhibitory concentration (FIC_50_) values were determined for seven different proportions of the compounds and artesunate, expressed in terms of IC_50_ equivalents, as mentioned before. FIC_50_ values from three independent experiments were modeled using nonlinear fitting and statistically compared to the additivity isobole. The absence of a statistical difference between the model and the additivity isobole indicated an additive drug combination, while statistically different curves indicated a synergistic (model below the additivity curve) or antagonistic (model above the additivity curve) drug combination.

### Antiplasmodial Activity Against Resistant Strains

The antiplasmodial activities of batzelladines L and F were evaluated against a representative panel of resistant strains. The panel included 3D7 (chloroquine-sensitive), Dd2 (resistant to chloroquine, mefloquine, and pyrimethamine), K1 (resistant to chloroquine, mefloquine, pyrimethamine, and sulfadoxine), Dd2^R^_DSM265 (resistant to DSM265, a PfDHODH inhibitor), and 3D7^R^_MMV848 (resistant to MMV848, a PfPI4K inhibitor). The evaluation of the IC_50_ values of the compounds against each resistant strain was carried out as previously described. After the determination of the IC_50_ value for each resistant strain (Figure S2), a resistance index (RI) was calculated by the ratio of the IC_50_^Resistant strain^ to IC_50_^3D7^. Of note, RI values >5 were considered indicative of cross-resistance.

### Antiplasmodial Activity Against Laboratory-Adapted Strains

The antiplasmodial activities of batzelladines L and F were evaluated against laboratory-adapted strains from Brazilian field isolates: BRPVH-39, BRPVH-41, and BRPVH-45. These isolates were originally sourced from blood samples collected from patients infected with in the region of Porto Velho, Rondônia. The evaluation of the IC_50_ values of the compounds against adapted strains was carried out previously. After the determination of the IC_50_ value for each laboratory-adapted strain, a ratio (R) was calculated by the ratio of the IC_50_^Adapted strain^ to IC_50_^3D7^.

### *Ex Vivo* Potency Against Infected Blood Samples—Schizont Maturation Test

Human blood samples were collected from patients in the region of Porto Velho, Rondônia. Schizont maturation assays were conducted following the established standard for schizont maturation testing (SMT).^30^ The assays were conducted at the Research Center for Tropical Medicine (CEPEM-RO) and complied with all relevant ethical regulations under the Ethics Committee CAAE 61442416.7.0000.0011. Briefly, blood samples were collected in heparinized tubes and then centrifuged (600*g*, 5 min). The RBCs were collected and filtered through a cellulose column aiming at removing residual white blood cells. The hematocrit was adjusted to 2%, and the resulting suspension was distributed onto 96-well plates with the prediluted compounds. Twelve dilutions of each compound were prepared in addition to untreated control wells. The plate was then incubated at 37 °C, with periodic monitoring of parasite morphology using thick smears. Once the control wells reached a morphological composition of ≥40% schizonts, thick smears were generated for all concentrations of compounds. These smears were then examined microscopically to assess their inhibitory activity. Schizont percentages for 200 observed parasites were analyzed for each compound concentration and used as a measure of inhibition relative to the control wells. IC_50_ values were calculated independently against each patient sample, and median IC_50_ values were used to represent compound potencies.

### *In Vivo* Assay Against 

A suppressive parasite growth test was performed in mice infected with NK65 strain (originally received from the New York University Medical School), as described previously,^34^ with some modifications. Briefly, adult female Swiss outbred mice (20 ± 2 g weight) were intraperitoneally inoculated with 1 × 10^5^ red blood cells infected with . The infected mice were maintained together for at least 2 h and then randomized into groups of 3 or 4 animals per cage, which were subsequently administered 50 mg/kg of each compound diluted in 3% (v/v) DMSO by oral gavage daily for 2 days. Two control groups were used in parallel: one was treated with CQ (20 mg/kg/day), and the other was treated with the vehicle, both for 3 days. Blood smears from mouse tails were prepared on days 5, 8, and 11 of experiment (total of 30 days of experiment) and then fixed with methanol, stained with Giemsa 10% (v/v), and examined under the microscope. The survival of the mice was carefully monitored throughout the entire duration of the experiment, extending up to the thirtieth day. Parasitemia was evaluated and the percent inhibition of parasite growth was calculated in relation to the untreated group (considered 100% growth) using the following equation

(C−T/C)×100

where C is the parasitemia in the control group and T is the parasitemia in the treated group. The use of laboratory animals was approved by the Ethics Committee for Animal Use of Universidade Federal do Estado de São Paulo, UNIFESP (CEUA N 6630080816). All institutional and national guidelines for the care and use of laboratory animals were followed.

### Isolation of Batzelladines F (**1**) and L (**2**)

The sponge was collected at Guarapari (Espirito Santo state) in 2017. Voucher specimens MNRJ 23075, 23076, and 23077. Sampling permit: SISBIO 10357-1 to E. Hajdu. A 400 g freeze-dried sample of the sponge was blended and extracted with MeOH (5 × 1 L). After solvent filtration, it was evaporated to 1.8 L, 200 mL of H_2_O was added, and the mixture was partitioned with hexane (3 × 2 L). The MeOH/H_2_O fraction was evaporated into 200 mL of H_2_O and partitioned with EtOAc (3 × 200 mL). The aqueous fraction was extracted with a mixture of XAD-2, XAD-4, and XAD-7 adsorption resins (10 g/100 mL) overnight. The mixture of resins was recovered by filtration and washed with H_2_O. The organic material adsorbed in the resins was desorbed with MeOH (2×) and with MeOH/acetone 50:50 (v/v) (2×) under sonication for 10 min each. The solution was evaporated resulting in the aqueous extract. Three fractions were obtained a resin-desorbed aqueous fraction (AqMA17; 21.8 g), an EtOAc fraction (AcMA17; 18.5 g) and a hexane fraction (HeMA17; 18 g). A 2.9 g sample of the AcMA17 fraction was solubilized in MeOH in an ultrasound bath and centrifuged for 10 min, and the supernatant was applied to a size-exclusion chromatography column (215 × 5 cm i.d.) containing Sephadex LH-20 as stationary phase. The separation was eluted with MeOH. Fractions of 13 mL were collected to give 165 fractions. After HPLC-PDA-ELSD-MS analysis of the separation fractions, these were pooled into 10 fractions: AcMA17_1, AcMA17_43, AcMA17_61, AcMA17_64, AcMA17_68, AcMA17_72, AcMA17_105, AcMA17_121, AcMa17_127, and AcMa17_137. Additional separations of the AcMA17 fraction (6.0 g) were performed using the same procedure, resulting in 13 final fractions: MA17s2_1, MA17s2_59, MA17s2_63, MA17s2_66, MA17s2_68, MA17s2_70, MA17s2_71, MA17s2_72, MA17s2_81, MA17s2_99, MA17s2_113, MA17s2_121, and MA17s2_143. Purification of the MA17s2_71 fraction by HPLC-DAD using a X-Terra column (Waters—C18, 7.8 × 150 mm, 5 μm), at a flow rate of 2.5 mL/min, and gradient elution of 40–70% MeOH:MeCN (1:1, v/v) and H_2_O in 20 min monitored at 215 nm, resulted the isolation of batzelladine L (**2**, 3.5 mg). HPLC using a X-Terra column (WatersGL Sciences Inc. – PhC18, 4.6 × 250 mm, 5 μm) at a flow rate of 1.0 mL/min, gradient elution of 40–60% of MeOH:MeCN (1:1, v/v), and H_2_O in 60 min monitored at 220 and 300 nm, resulted in the isolation of batzelladine F (**1**, 1.4 mg). Additional amounts of **1** (16 mg) and **2** (43 mg) were obtained from the fraction AcMA17_43, using an InertSustain column (GL Sciences Inc.—Ph, 4.6 × 250 mm, 5 μm) at a flow rate of 1.0 mL/min and gradient elution of 25–40% MeCN and H_2_O for 50 min monitored at 220 and 300 nm. The gradient consisted in three steps of 5% per 1 min each increment, sustained isocratic for 3 min in 30 and 35% of MeCN, when it was raised to 40% per 1 min and kept isocratic from 12 to 20 min. Finally, the system was washed with 60% MeCN from 20 to 30 min returning to equilibrium at 25% MeCN from 30 to 40 min. Additional amounts of **1** were obtained by HPLC using a X-Terra column (Waters—C18, 4.6 × 250 mm, 5 μm) at a flow rate of 1.0 mL/min, gradient elution of 34–54% of MeOH:MeCN (1:1, v/v), and H_2_O in 60 min, monitored at 265 and 313 nm.

## Supplementary Material



## Data Availability

The data underlying this study are available in the published article and its online Supporting Information.

## Notes

Concentration–response curve of batzelladines F (**1**) and L (**2**); spectroscopic and spectrometric analysis of **1** and **2**; fragmentation proposal for compounds **1** and **2**; general experimental and molecular modeling procedures (PDF)
