# Supplementary material for: Marine Guanidine Alkaloids Inhibit Malaria Parasites Development in In Vitro, In Vivo and Ex Vivo Assays
Source: ACS Infect Dis. 2025 Apr 15;11(7):1854–67. doi: 10.1021/acsinfecdis.4c00714 (PMC12261324; doi:10.1021/acsinfecdis.4c00714)
Supplement: Supplementary file 1 [file id4c00714_si_001.pdf]

Supporting Information

For

Marine Guanidine Alkaloids Inhibit Malaria Parasites Development in *In vitro, In Vivo* and *Ex Vivo* Assays

Giovana Rossi Mendes,[†#] Anderson L. Noronha,[‡#] Igor M. R. Moura,[†] Natália Menezes Moreira,[†] Vinícius Bonatto,[†] Camila S. Barbosa,[†] Sarah El Chamy Maluf,[†] Guilherme Eduardo de Souza,[†] Marcelo Rodrigues de Amorim,[‡] Anna Caroline Campos Aguiar,[†§] Fabio C. Cruz,[⊥] Amália dos Santos Ferreira,[‖] Carolina B. G. Teles,[‖] Dhelio B. Pereira,[▽] Eduardo Hajdu,[O] Antonio G. Ferreira,[¶] Roberto G. S. Berlinck,[‡*] Rafael Victorio Carvalho Guido[†*]

[†]São Carlos Institute of Physics, University of Sao Paulo, CEP 13563-120, São Carlos, SP, Brazil.
[‡]Instituto de Química de São Carlos, Universidade de São Paulo, CEP 13560-970, São Carlos, SP, Brazil
[§]Department of Microbiology, Immunology and Parasitology, Federal University of São Paulo, CEP 04023-062, São Paulo, SP, Brazil.
[⊥]Department of Pharmacology, Federal University of São Paulo, CEP 04023-062, São Paulo, SP Brazil.
[‖]Oswaldo Cruz Foundation, Leishmaniasis and Malaria Bioassay Platform, CEP 76812-245, Porto Velho, Rondônia, Brazil.
[▽]Research Center in Tropical Medicine of Rondônia, CEP 76812-329, Porto Velho, RO, Brazil.
[O]Museu Nacional, Universidade Federal do Rio de Janeiro, CEP 20940-040, Rio de Janeiro, RJ, Brazil.
[¶]Departamento de Química, Universidade Federal de São Carlos, CEP 13565-905, São Carlos, SP, Brazil

#These authors contributed equally.

*****Corresponding authors

E-mail: rgsberlinck@iqsc.usp.br ORCID: https://orcid.org/0000-0003-0118-2523

E-mail: rvcguido@usp.br ORCID: https://orcid.org/0000-0002-7187-0818

List of Supplementary Figures

Figure S1. Concentration-response curve of (**a**) batzelladine L and (**b**) batzelladine F against *P. falciparum* 3D7 strain, chloroquine-sensitive. Concentration-response curve of (**c**) batzelladine L and (**d**) batzelladine F against human hepatocellular carcinoma cells (HepG2 cell line). S5

Figure S2. Concentration-response curves of the standard antimalarials (artesunate, pyrimethamine, DSM265 and MMV692848) and batzelladines L and F against a panel of resistant strains (Dd2, K1, Dd2^R_DSM265 and 3D7^R_MMV848). S6

Figure S3. Assessment of the combination of batzelladine L with chloroquine. The red region and red dots represent the experimental data, while the black line and gray region indicate the additivity curve. (A) Isobologram of batzelladine L combined with chloroquine. (B) Statistical analysis for the combination of batzelladine L with chloroquine. These represent the ∑FIC50 values of three independent experiments (p-value < 0.05 shows statistical difference between the experimental data and the additivity isobole). S7

Figure S4. (A) Schematic representation of the speed of action assay. The blue arrows represent the drug removal for each time of drug exposure. (**B**) Schematic representation of the extended speed of action assay. The orange arrows represent the time points when plates were assessed using the SYBR Green I assay to determine the inhibitory activity. S8

Figure S5. Schematic representation of the stage-specificity inhibition assay. The green arrows represent the drug pressure for each time of drug exposure and the blue arrows represent the drug removal. S8

Figure S6. ^{1}H NMR spectrum of **1** (600 MHz, CD_3OD). S9

Figure S7. ^{13}C NMR spectrum of **1** (150 MHz,CD_3OD). S10

Figure S8. *g*COSY NMR spectrum of **1** (600 MHz, CD_3OD). S11

Figure S9. *g*HSQC NMR spectrum of **1** (^{1}H: 600 MHz, ^{13}C: 150 MHz, CD_3OD) S12

Figure S10. *g*HMBC NMR spectrum of **1** (^{1}H: 600 MHz, ^{13}C: 150 MHz, CD_3OD). . S13

Figure S11. Total ion chromatogram (TIC) of **1** by HPLC-MS. Column C_{18}, Xterra (Waters, 4.6 mm × 250 mm, 5 µm). S14

Figure S12. LR-ESI-MS spectrum of **1** in positive ionization mode by HPLC-MS. .. S15

Figure S13. HR-ESI-MS spectrum of **1** in positive ionization mode by UPLC-ESI-qTOF-MS. S16

Figure S14. HR-ESI-qTOF-MS/MS spectrum of **1** in positive ionization mode (35 eV). S17
Figure S15. Fragmentation proposal for compound **1** by HR-ESI-qTOF-MS/MS in positive ionization mode........ S18
Figure S16. ^{1}H NMR spectrum of **2** (600 MHz, CD_3OD)........ S19
Figure S17. ^{13}C NMR spectrum of **2** (150 MHz, CD_3OD)........ S20
Figure S18. *g*HSQC NMR spectrum of **2** (^{1}H: 600 MHz, ^{13}C: 150 MHz, CD_3OD).... S21
Figure S19. *g*HMBC NMR spectrum of **2** (^{1}H: 600 MHz, ^{13}C: 150 MHz, CD_3OD). . S22
Figure S20. *g*COSY NMR spectrum of **2** (600 MHz, CD_3OD). S23
Figure S21. Total ion chromatogram (TIC) of **2** by HPLC-MS. Column C_{18} Xterra (Waters, 4.6 mm × 250 mm, 5 µm). S24
Figure S22. LR-ESI-MS spectrum of **2** in positive ionization mode by HPLC-MS. .. S25
Figure S23. HR-ESI-MS spectrum of **2** in positive ionization mode by UPLC-ESI-qTOF-MS. S26
Figure S24. HR-ESI-qTOF-MS/MS spectrum of **2** in positive ionization mode. S27
Figure S25. Fragmentation proposal for compound **2** by HR-ESI-qTOF-MS/MS in positive ionization mode........ S28
Figure S26. MD analysis of batzelladine F-*Pf*ENT1 over 1µs of simulation**. A)** RMSD of the protein-ligand complex; **B)** RMSD of the ligand; **C)** RMSF of the C_α atoms throughout the simulation........ S29
Figure S27. MD analysis of batzelladine L-*Pf*ENT1 over 1µs of simulation**. A)** RMSD of the protein-ligand complex; **B)** RMSD of the ligand; **C)** RMSF of the C_α atoms throughout the simulation........ S29
Figure S28. Distance between OE2 of Glu618 and H63 of the protonated tricyclic guanidine moiety in batzelladine F over 1µs of simulation. S31
Figure S29. A) Binding mode of BatF interacting with Gln135 through a water bridge; **B)** Frequency of the water bridge between the carbonyl O1 of batzelladine F and the side chain of Gln135 (C=O or NH_2) over 1µs of simulation. A value of 0 indicates no water bridge formation, while a value of 1 indicates the presence of a water bridge between BatF and Gln135........ S31
Figure S30. Distance between HG of Ser154 and the carbonyl O1 of batzelladine L over 1µs of simulation. S32
Figure S31. Distance between OE2 of Glu618 and H67 of the protonated tricyclic guanidine moiety in batzelladine L over 1µs of simulation. S32

List of Supplementary Tables

Table S1. Comparison of ^{1}H (600 Hz) and ^{13}C (150 Hz) NMR data for **1** with the data reported in the literature for batzelladine F………………………………………..S33

Table S2. Comparison of ^{1}H (600 Hz) and ^{13}C (150 Hz) NMR data for **2** with the data reported in the literature for batzelladine L……………………………………………S35

Experimental

Molecular Modelling Procedures S33

General Experimental Procedures S38

References S40

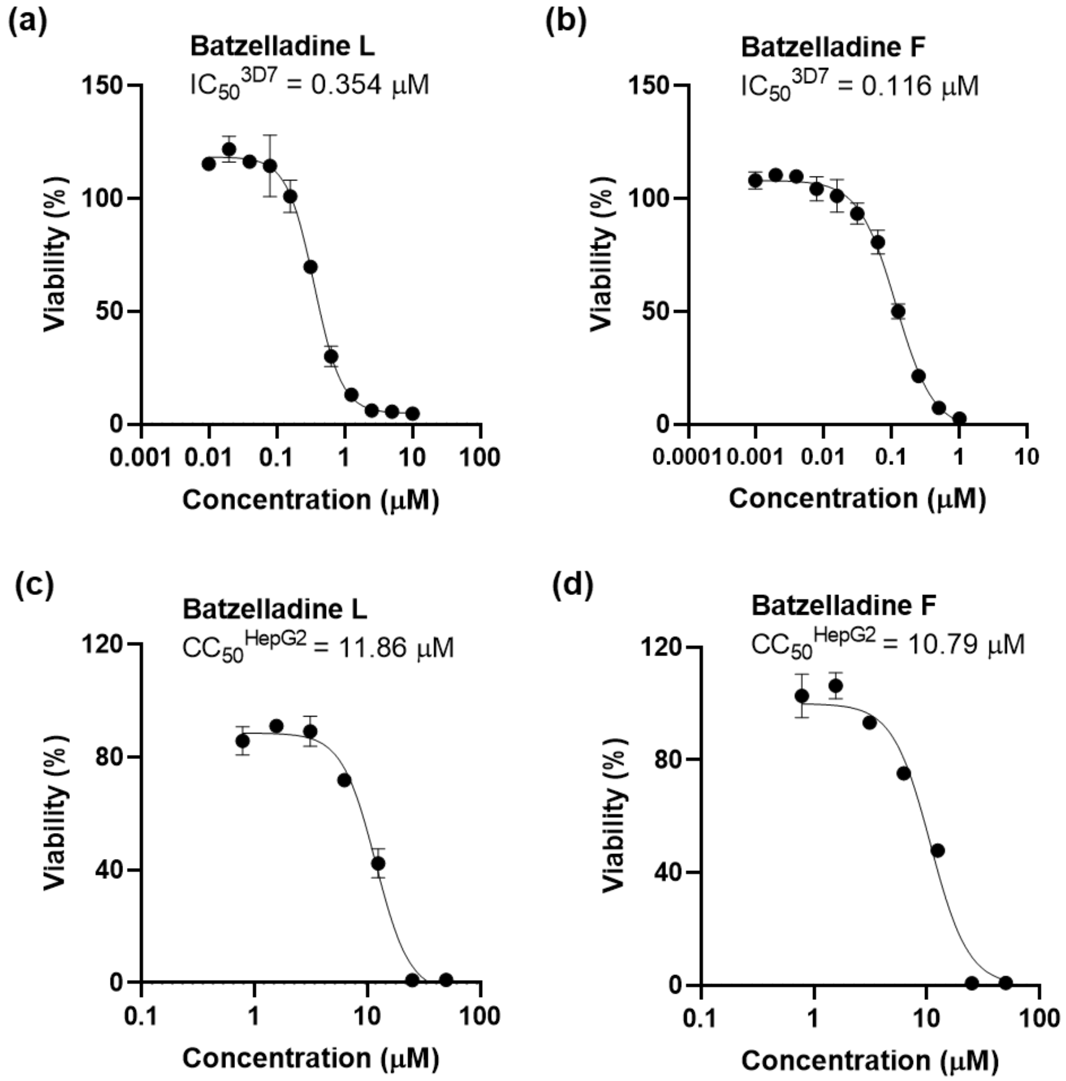

Figure S1. Concentration-response curve of (**a**) batzelladine L and (**b**) batzelladine F against *P. falciparum* 3D7 strain, chloroquine-sensitive. Concentration-response curve of (**c**) batzelladine L and (**d**) batzelladine F against human hepatocellular carcinoma cells (HepG2 cell line).

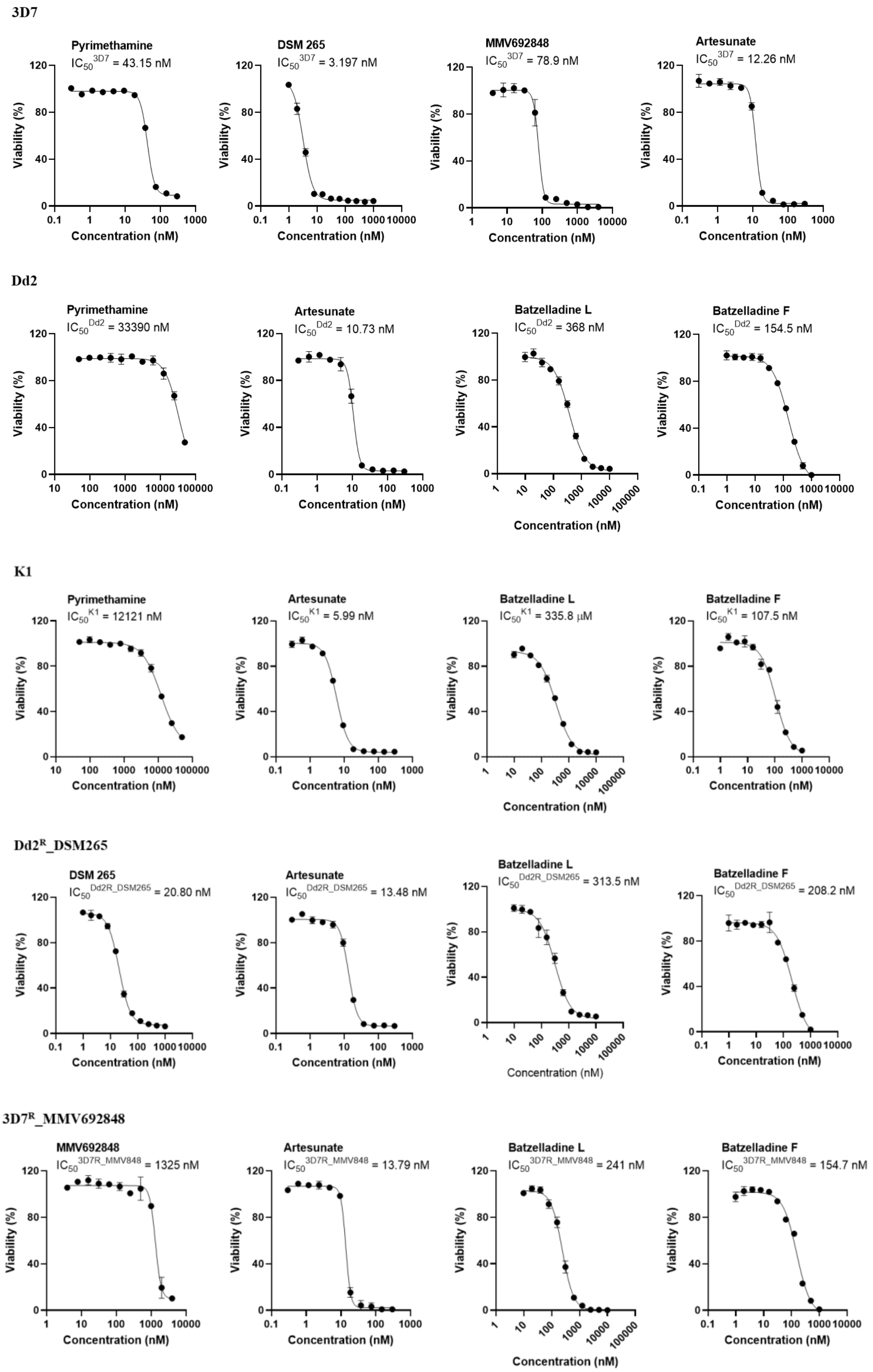

Figure S2. Concentration-response curves of the standard antimalarials (artesunate, pyrimethamine, DSM265 and MMV692848) and batzelladines L and F against a panel of resistant strains (Dd2, K1, Dd2^{R}_DSM265 and 3D7^{R}_MMV848).

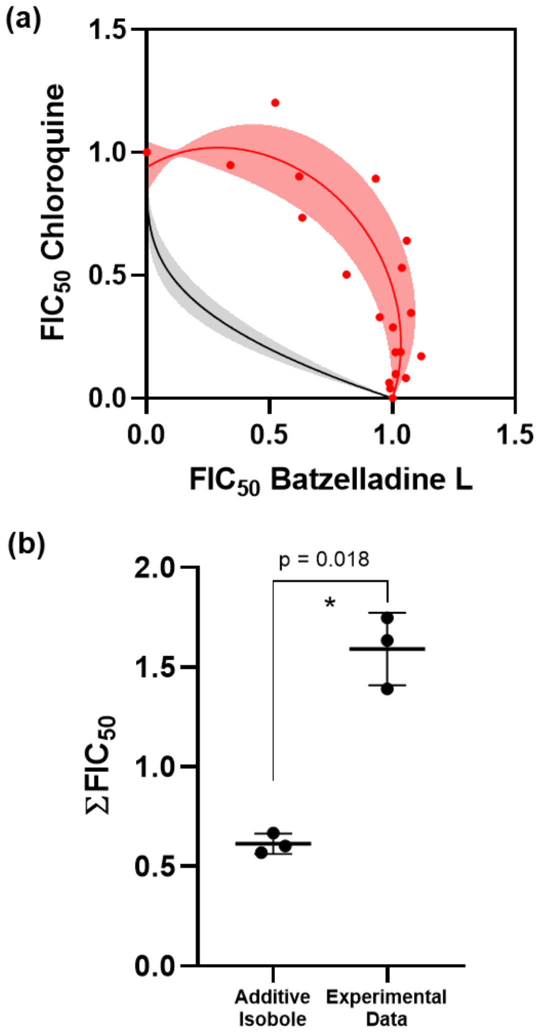

Figure S3. Assessment of the combination of batzelladine L with chloroquine. The red region and red dots represent the experimental data, while the black line and gray region indicate the additivity curve. (A) Isobologram of batzelladine L combined with chloroquine. (B) Statistical analysis for the combination of batzelladine L with chloroquine. These represent the ∑FIC50 values of three independent experiments (p-value < 0.05 shows statistical difference between the experimental data and the additivity isobole).

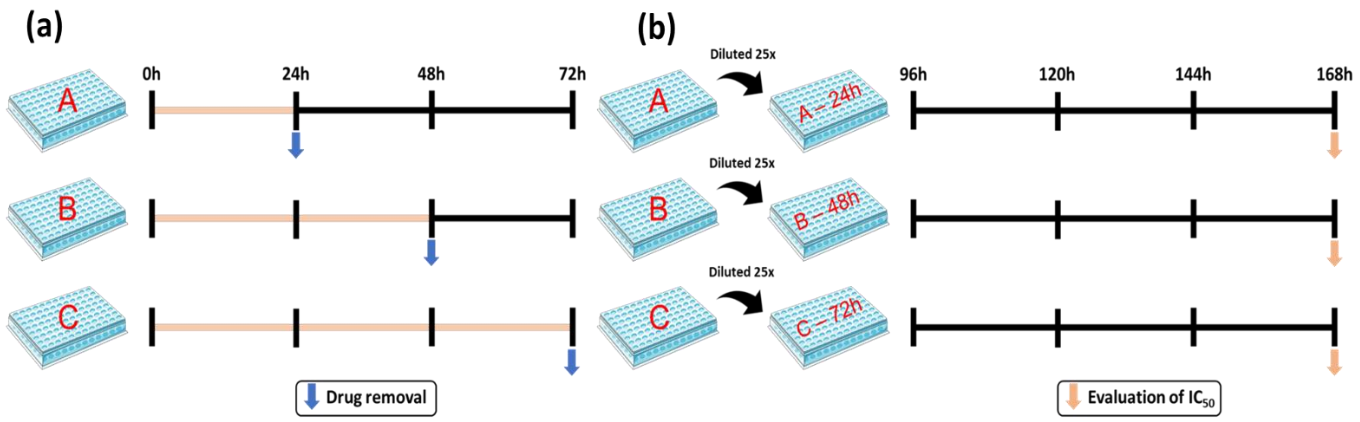

Figure S4. (A) Schematic representation of the speed of action assay. The blue arrows represent the drug removal for each time of drug exposure. (**B**) Schematic representation of the extended speed of action assay. The orange arrows represent the time points when plates were assessed using the SYBR Green I assay to determine the inhibitory activity.

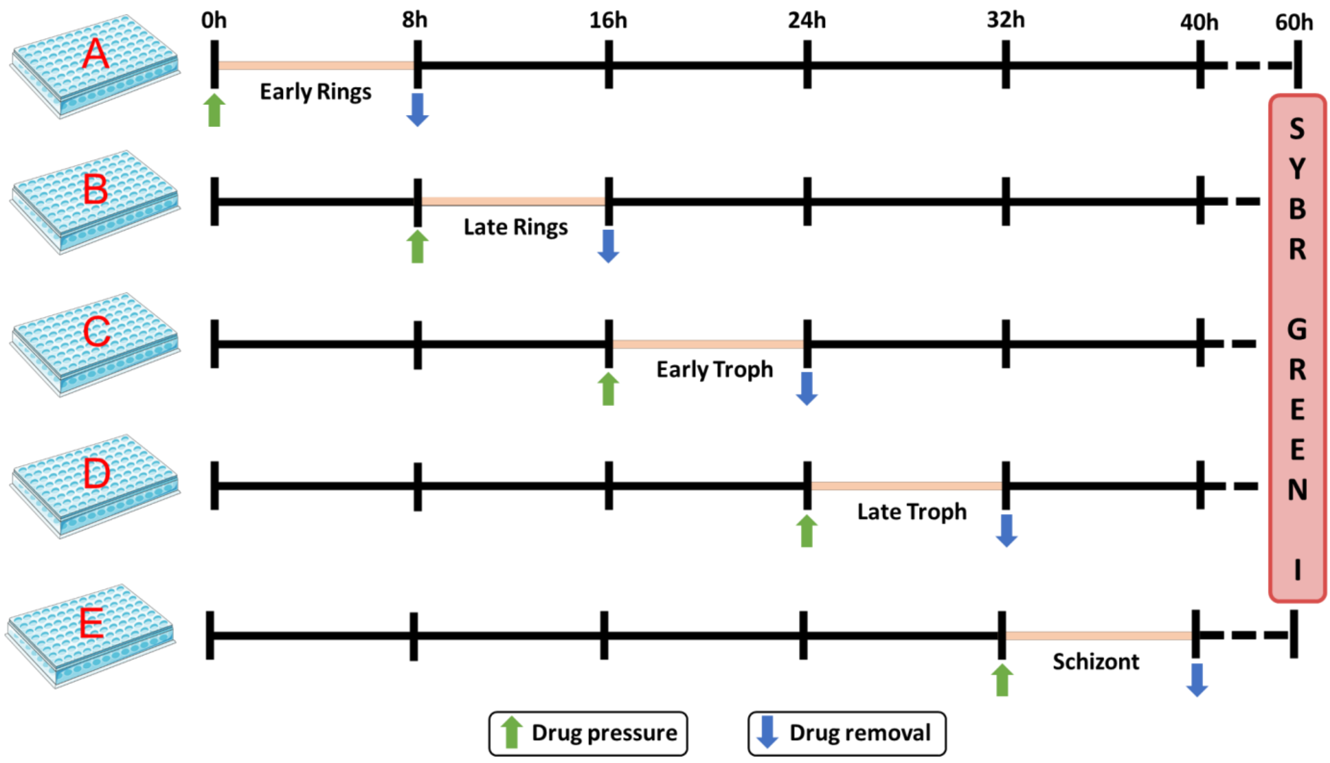

Figure S5. Schematic representation of the stage-specificity inhibition assay. The green arrows represent the drug pressure for each time of drug exposure and the blue arrows represent the drug removal.

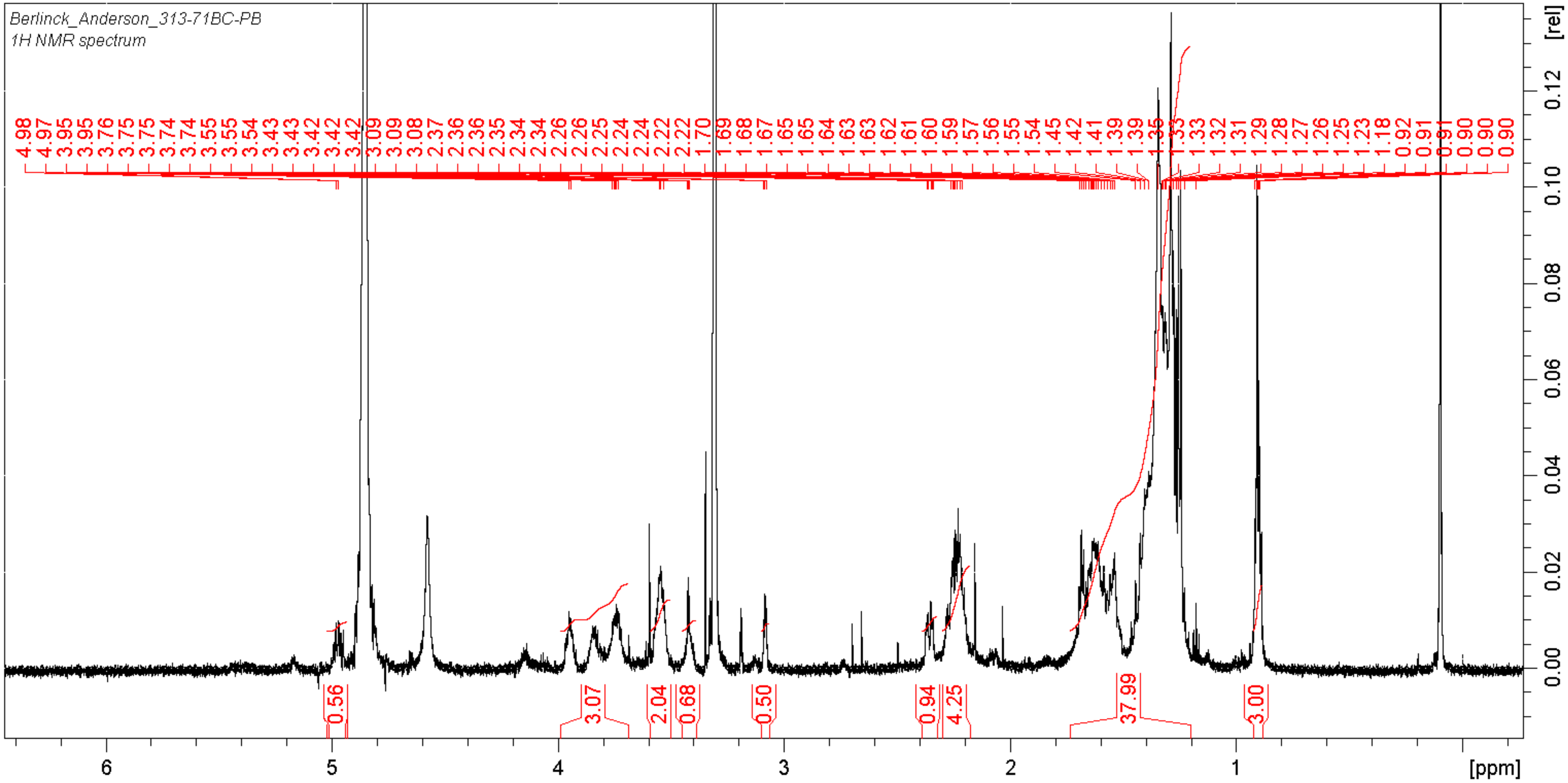

Figure S6. ^{1}H NMR spectrum of **1** (600 MHz, CD_3OD).

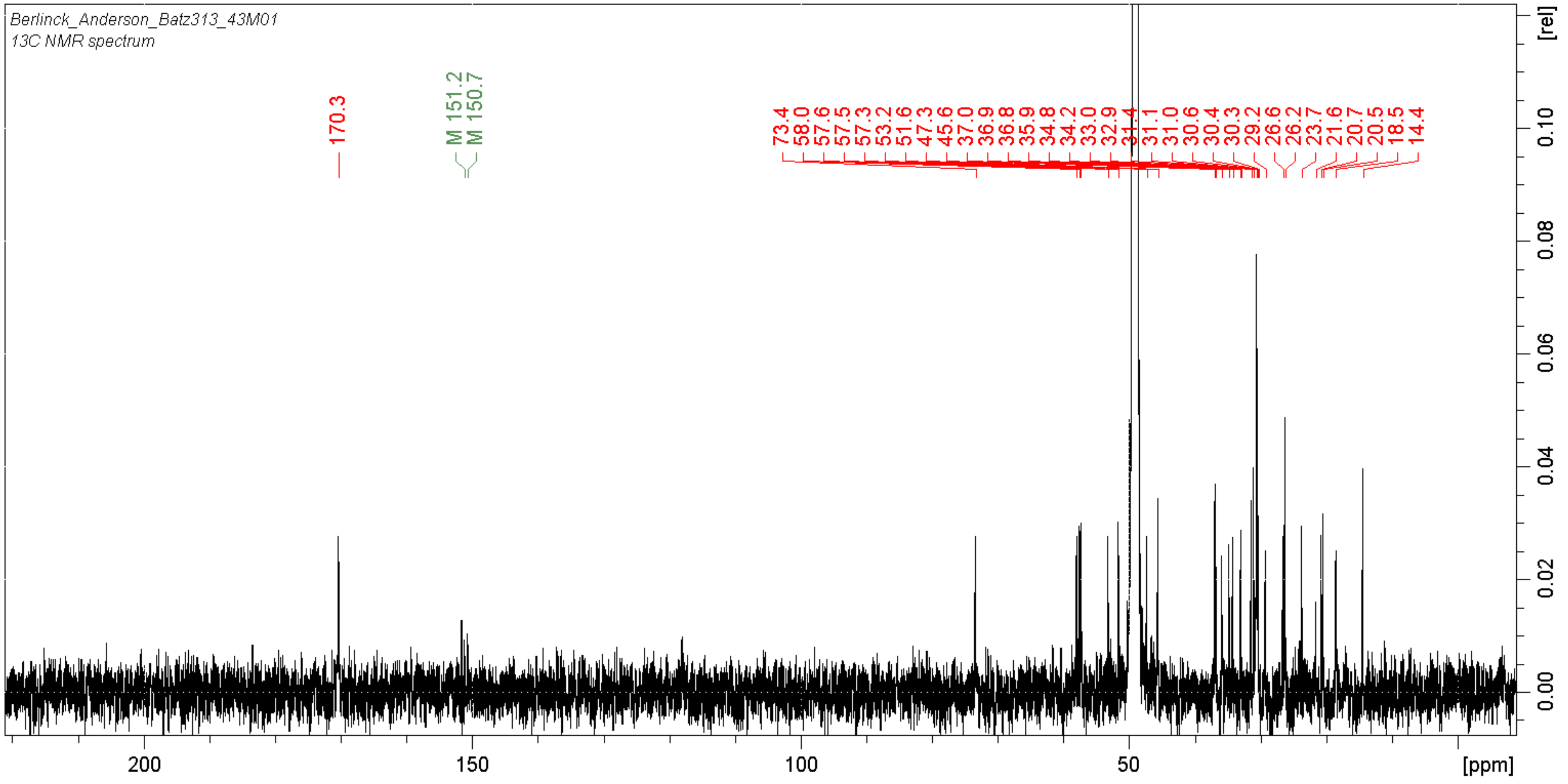

Figure S7. ^{13}C NMR spectrum of **1** (150 MHz,CD_3OD).

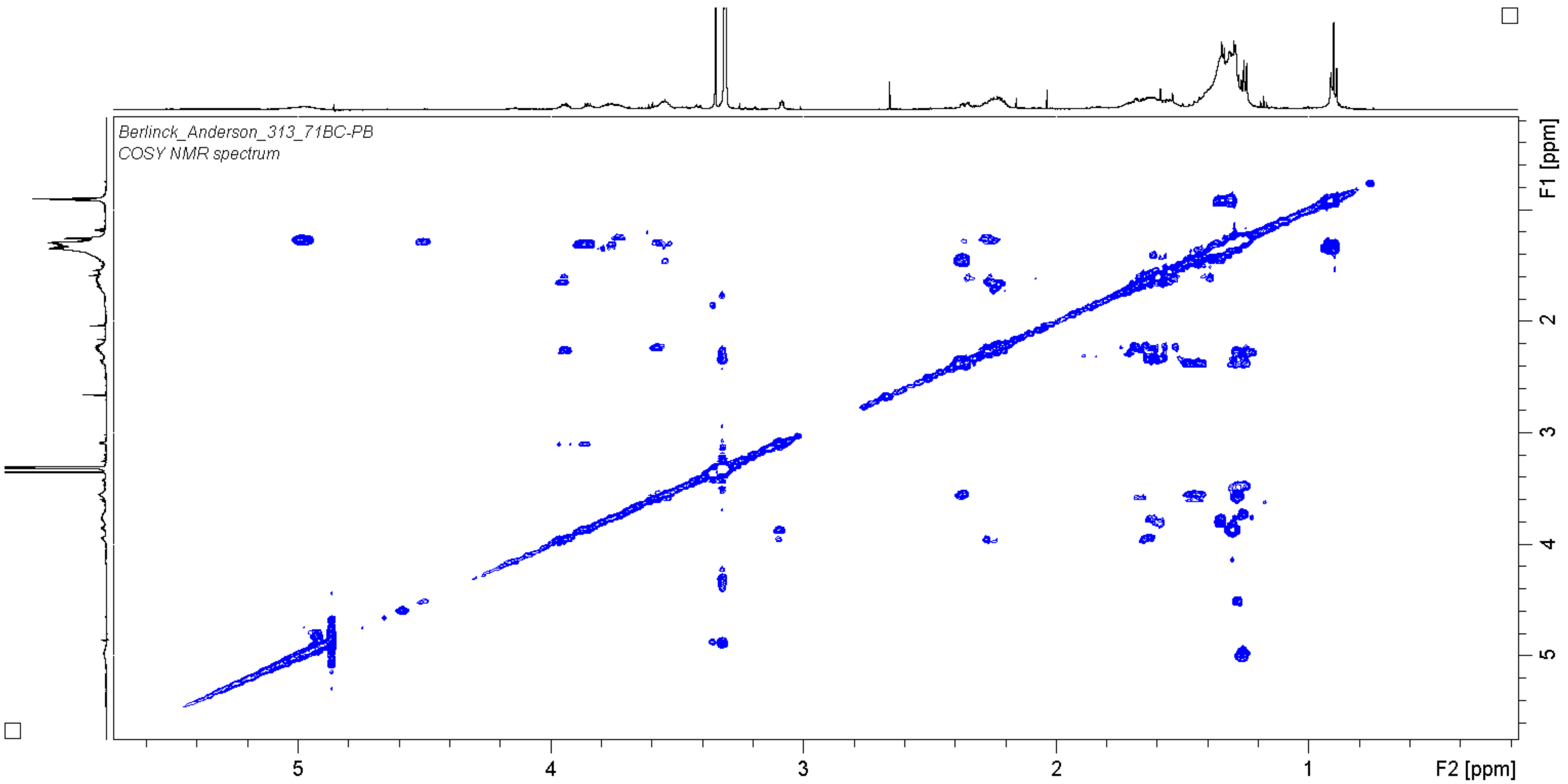

Figure S8. *g*COSY NMR spectrum of **1** (600 MHz, CD_3OD).

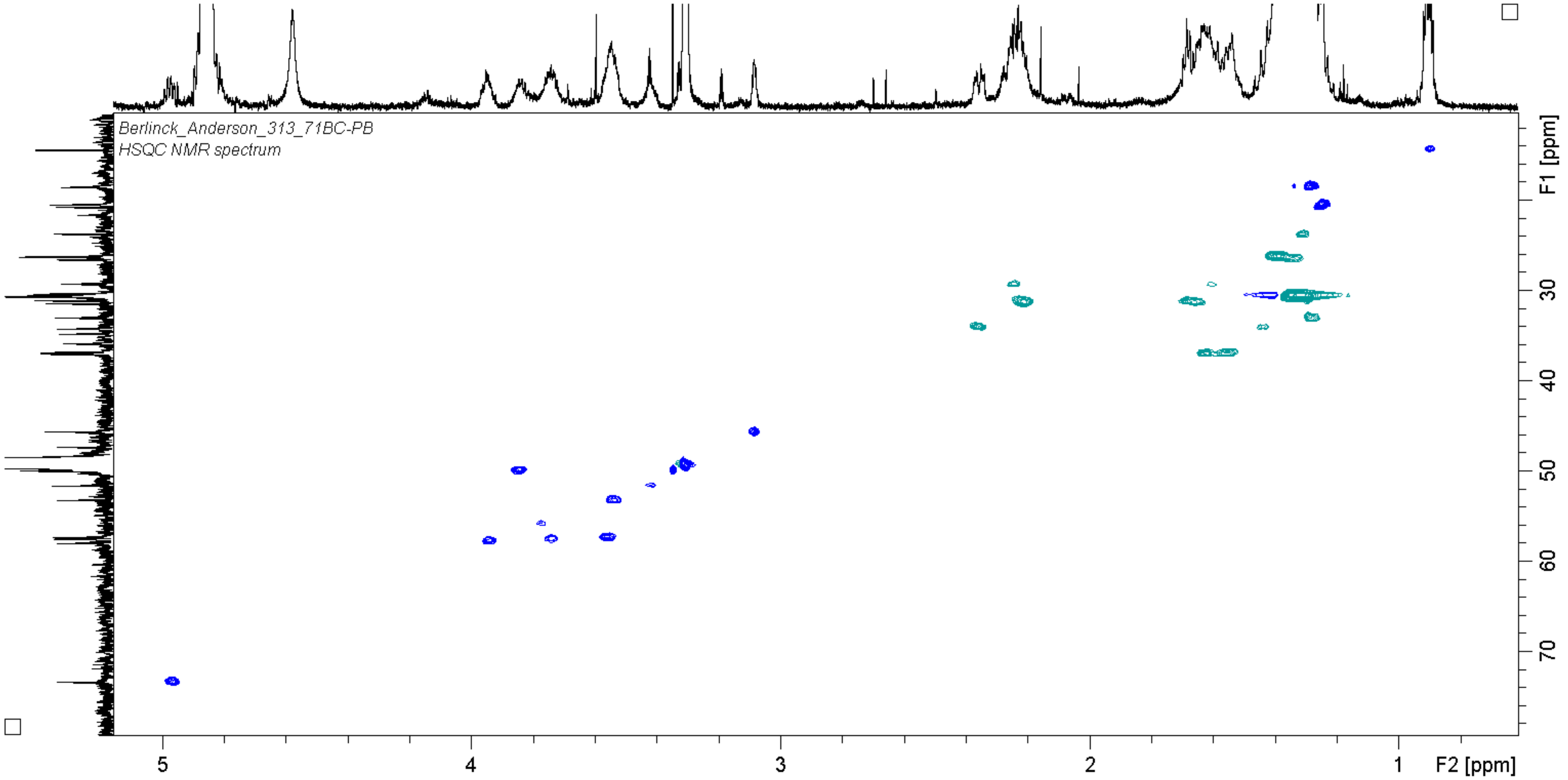

Figure S9. *g*HSQC NMR spectrum of **1** (^{1}H: 600 MHz, ^{13}C: 150 MHz, CD_3OD).

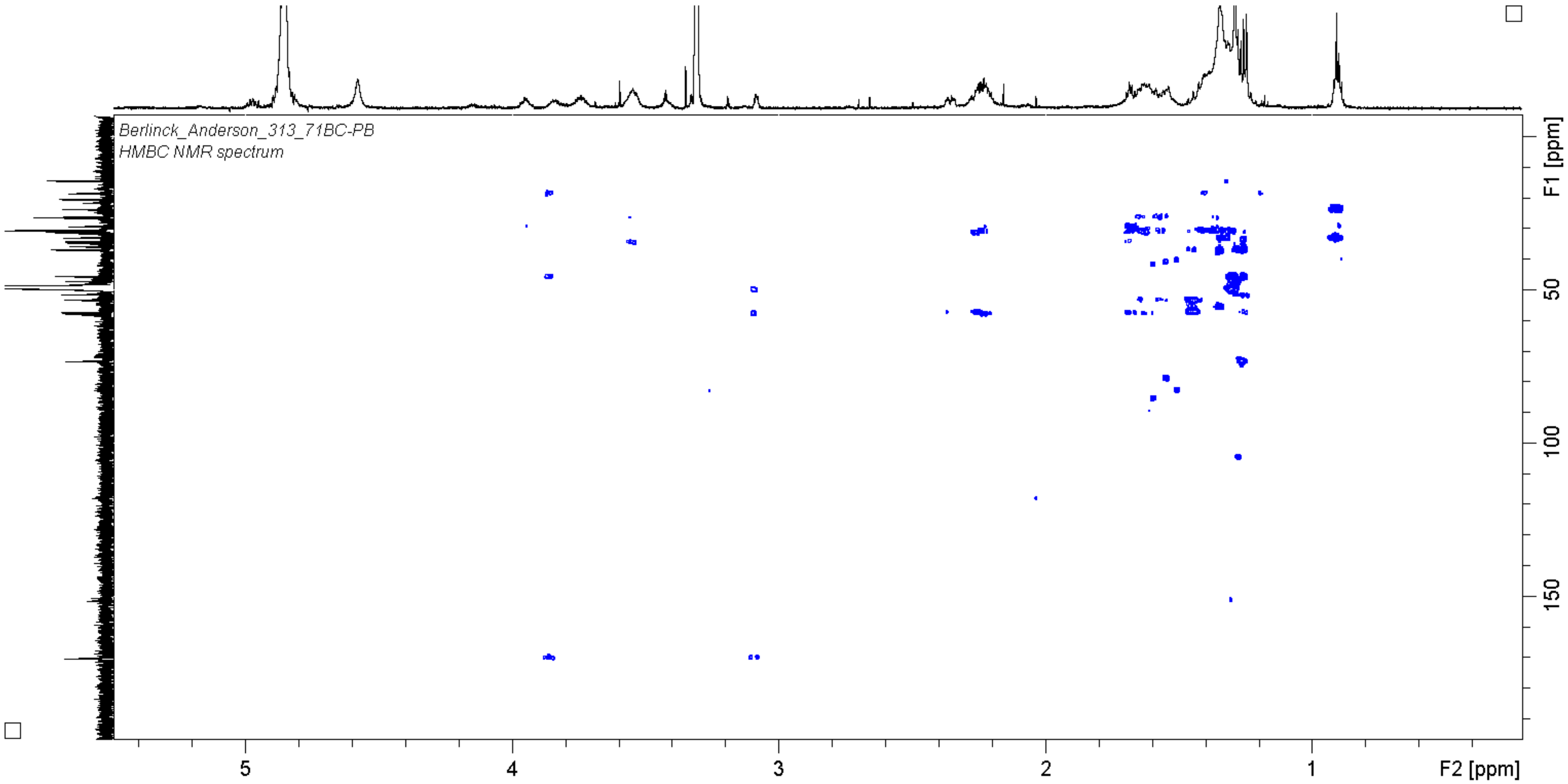

Figure S10. *g*HMBC NMR spectrum of **1** (^{1}H: 600 MHz, ^{13}C: 150 MHz, CD_3OD).

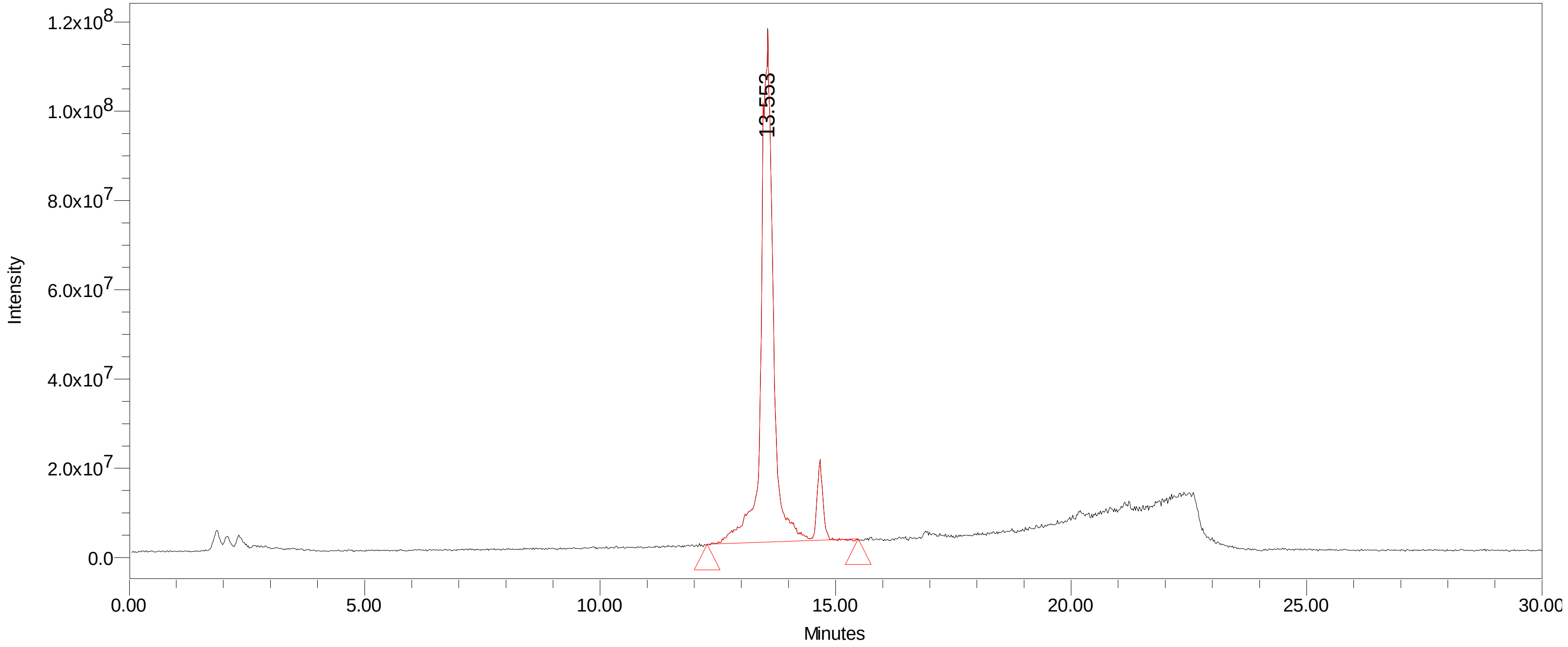

Figure S11. Total ion chromatogram (TIC) of **1** by HPLC-MS. Column C_{18}, Xterra (Waters, 4.6 mm × 250 mm, 5 µm).

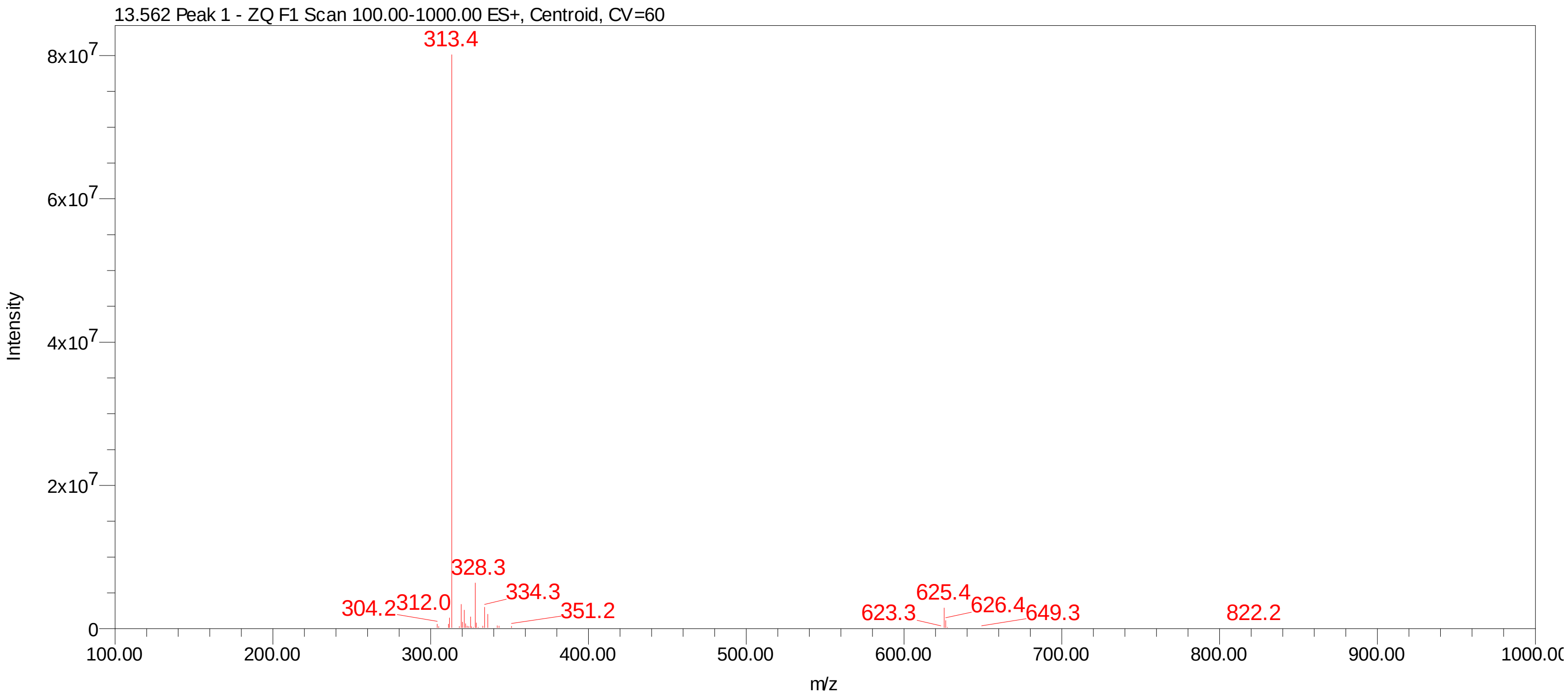

Figure S12. LR-ESI-MS spectrum of **1** in positive ionization mode by HPLC-MS.

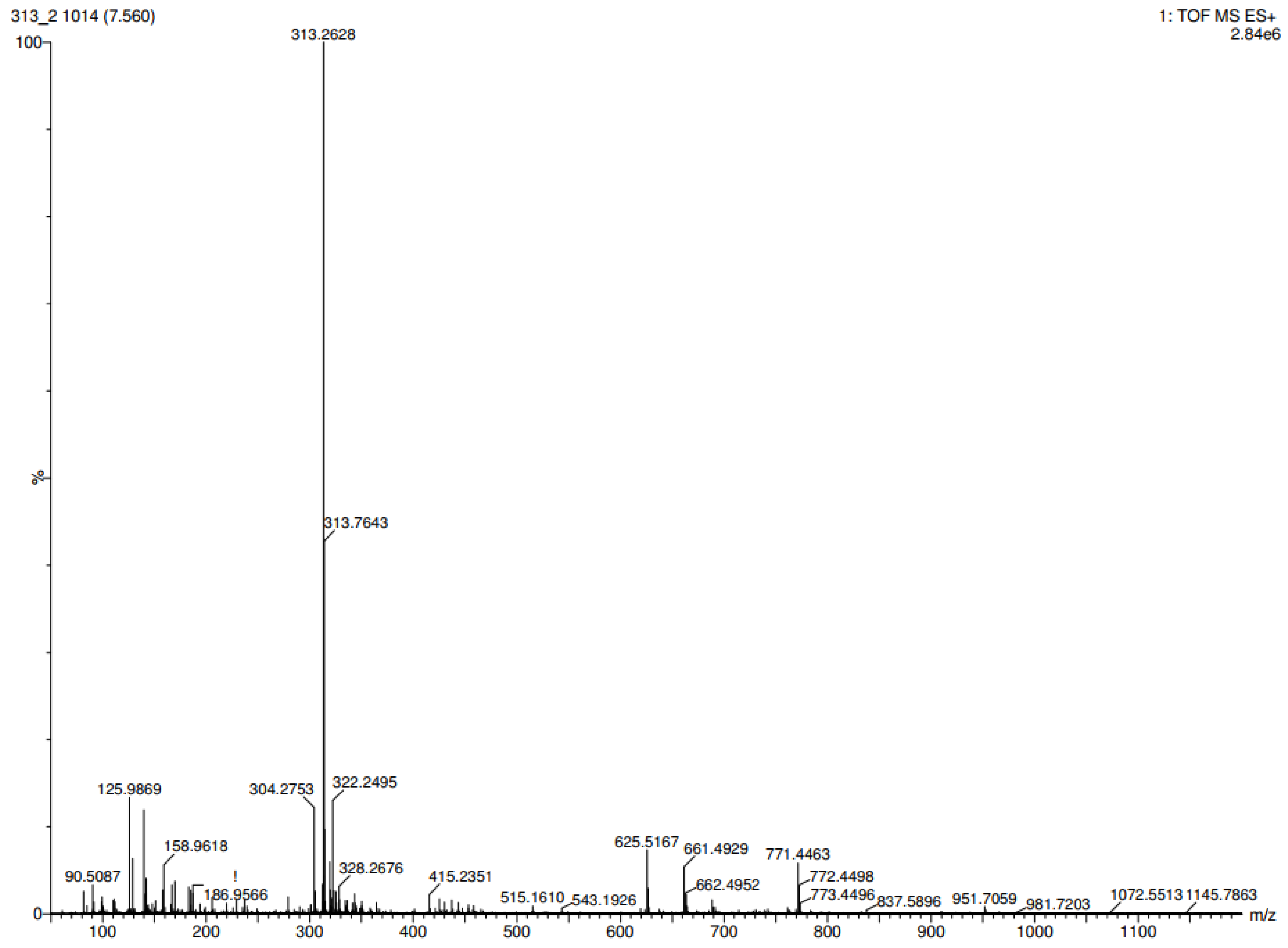

Figure S13. HR-ESI-MS spectrum of **1** in positive ionization mode by UPLC-ESI-qTOF-MS.

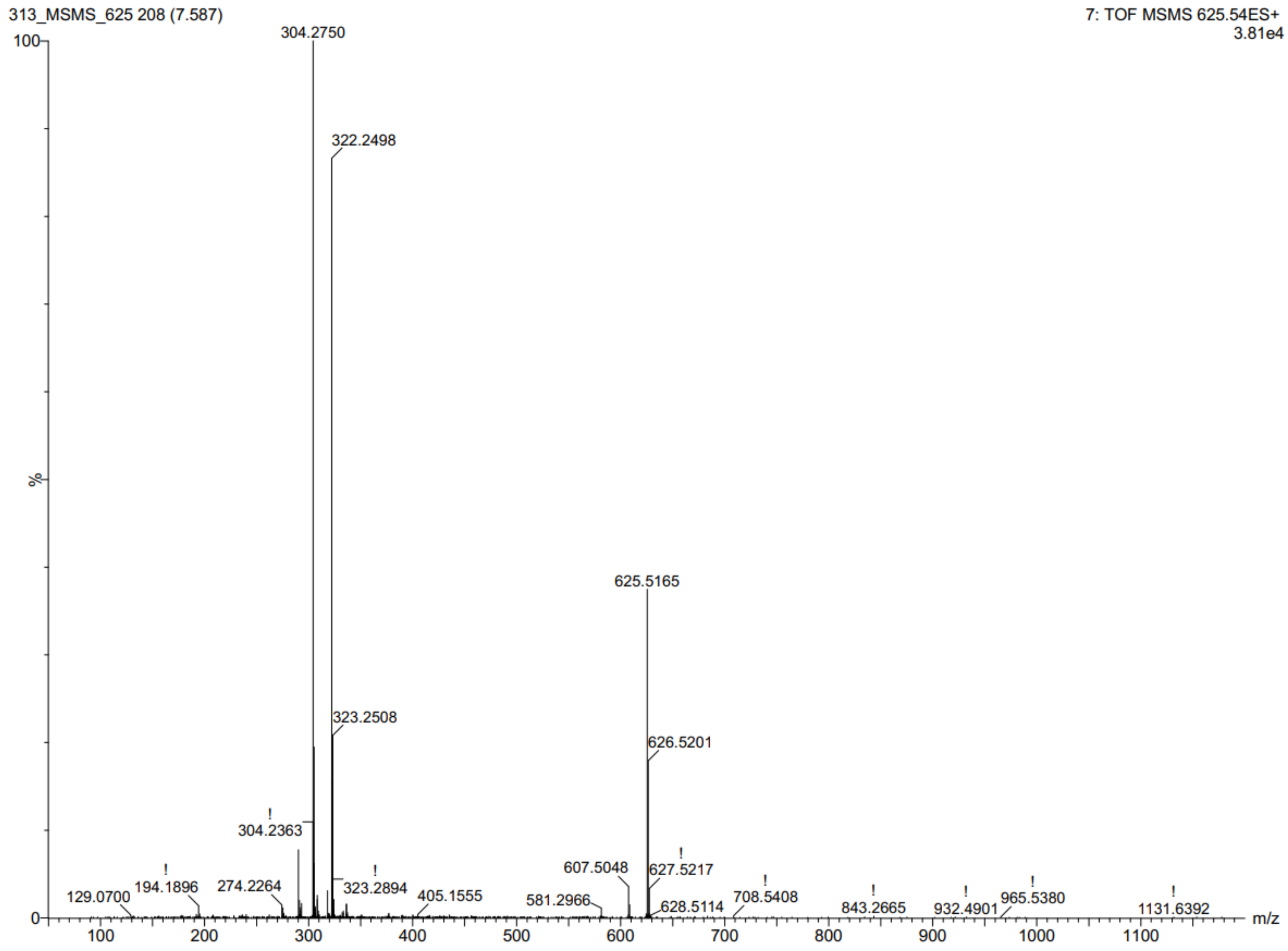

Figure S14. HR-ESI-qTOF-MS/MS spectrum of **1** in positive ionization mode (35 eV).

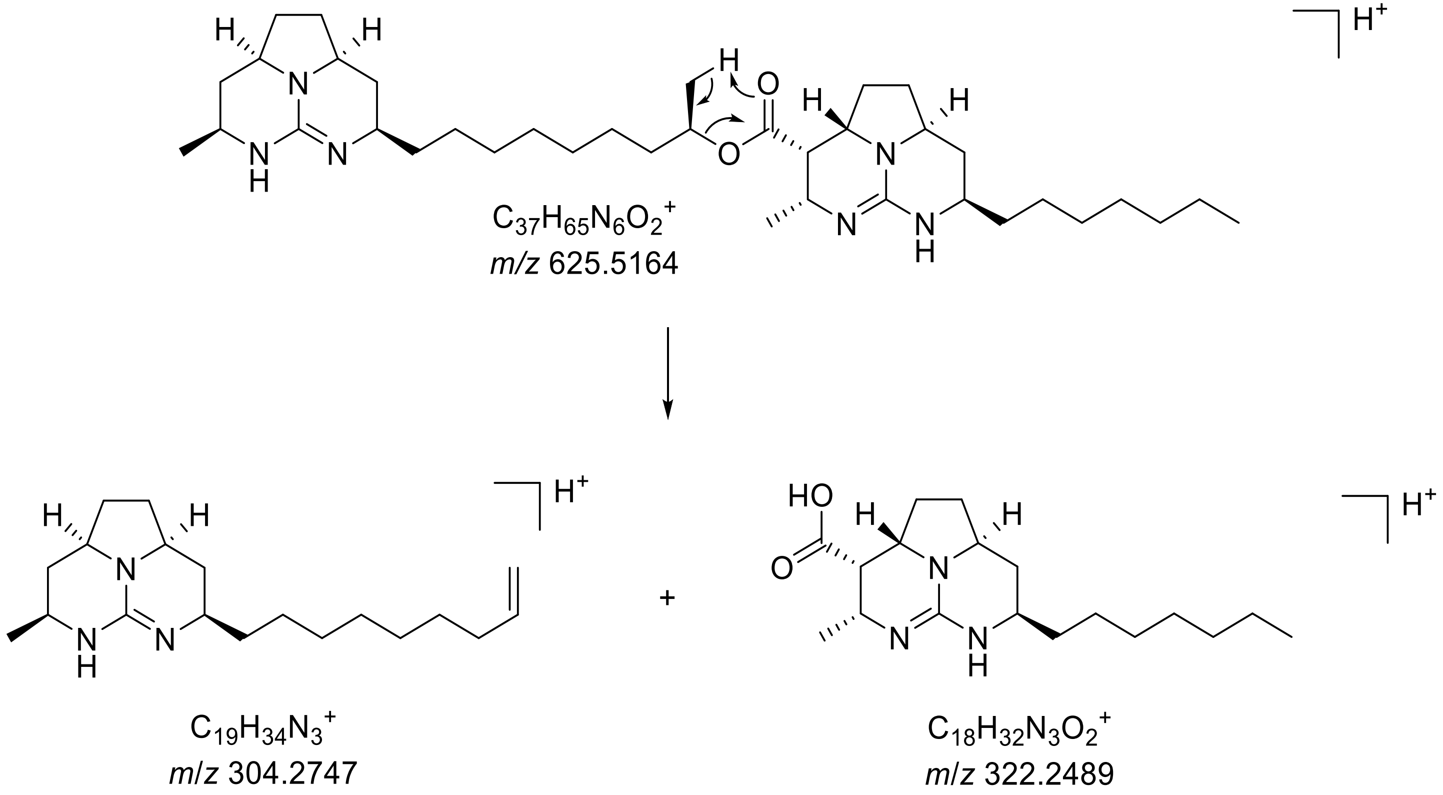

Figure S15. Fragmentation proposal for compound **1** by HR-ESI-qTOF-MS/MS in positive ionization mode.

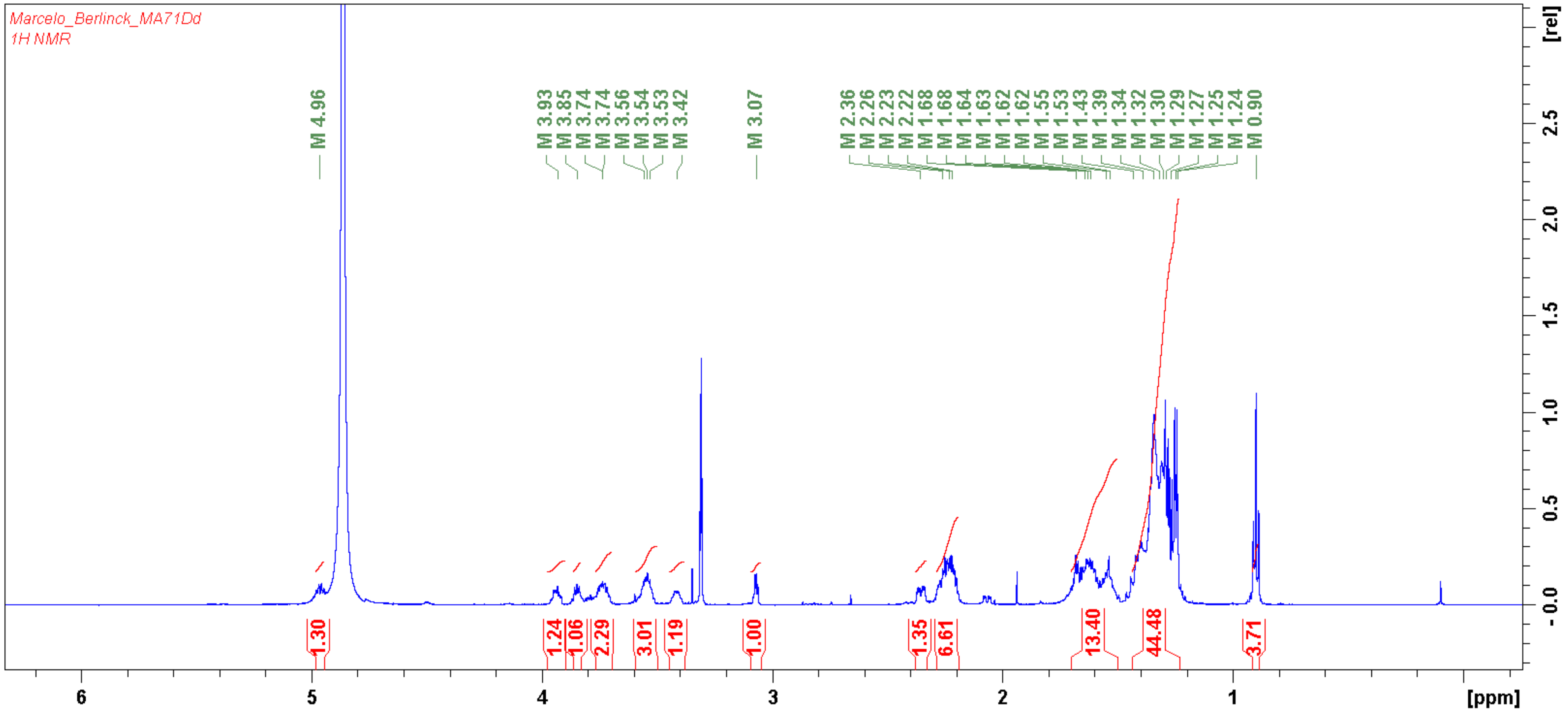

Figure S16. ^{1}H NMR spectrum of **2** (600 MHz, CD_3OD).

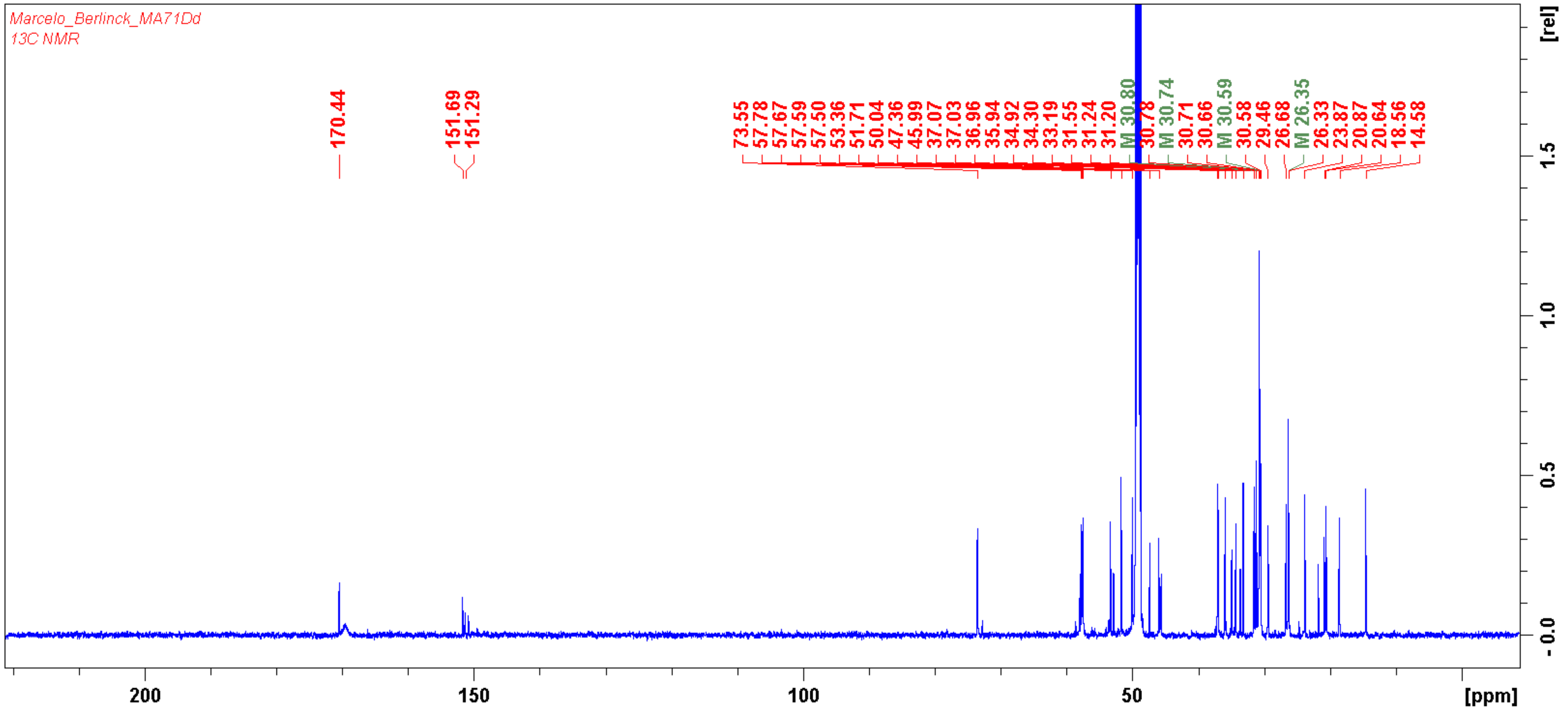

Figure S17. ^{13}C NMR spectrum of **2** (150 MHz, CD_3OD).

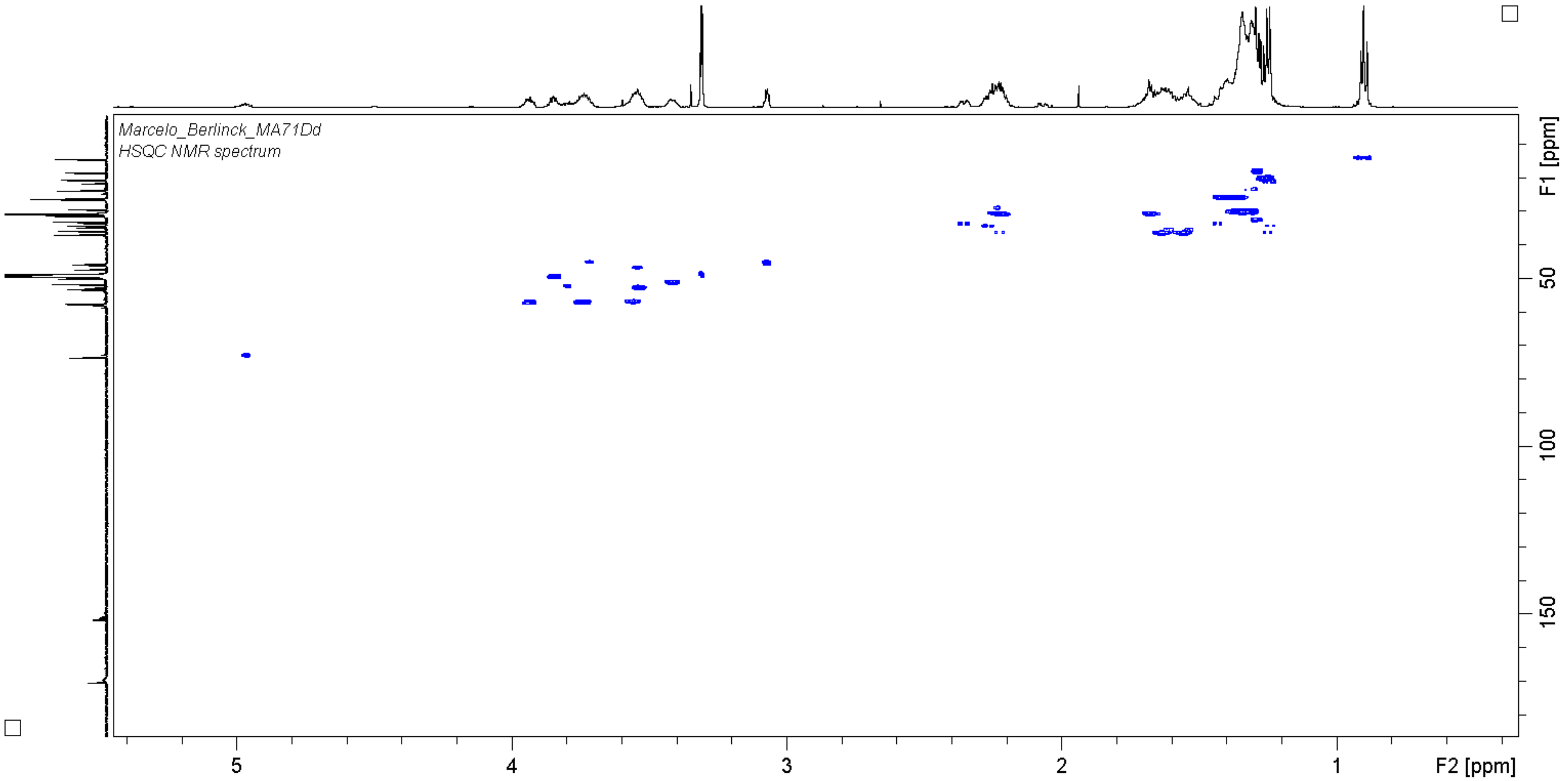

Figure S18. *g*HSQC NMR spectrum of **2** (^{1}H: 600 MHz, ^{13}C: 150 MHz, CD_3OD).

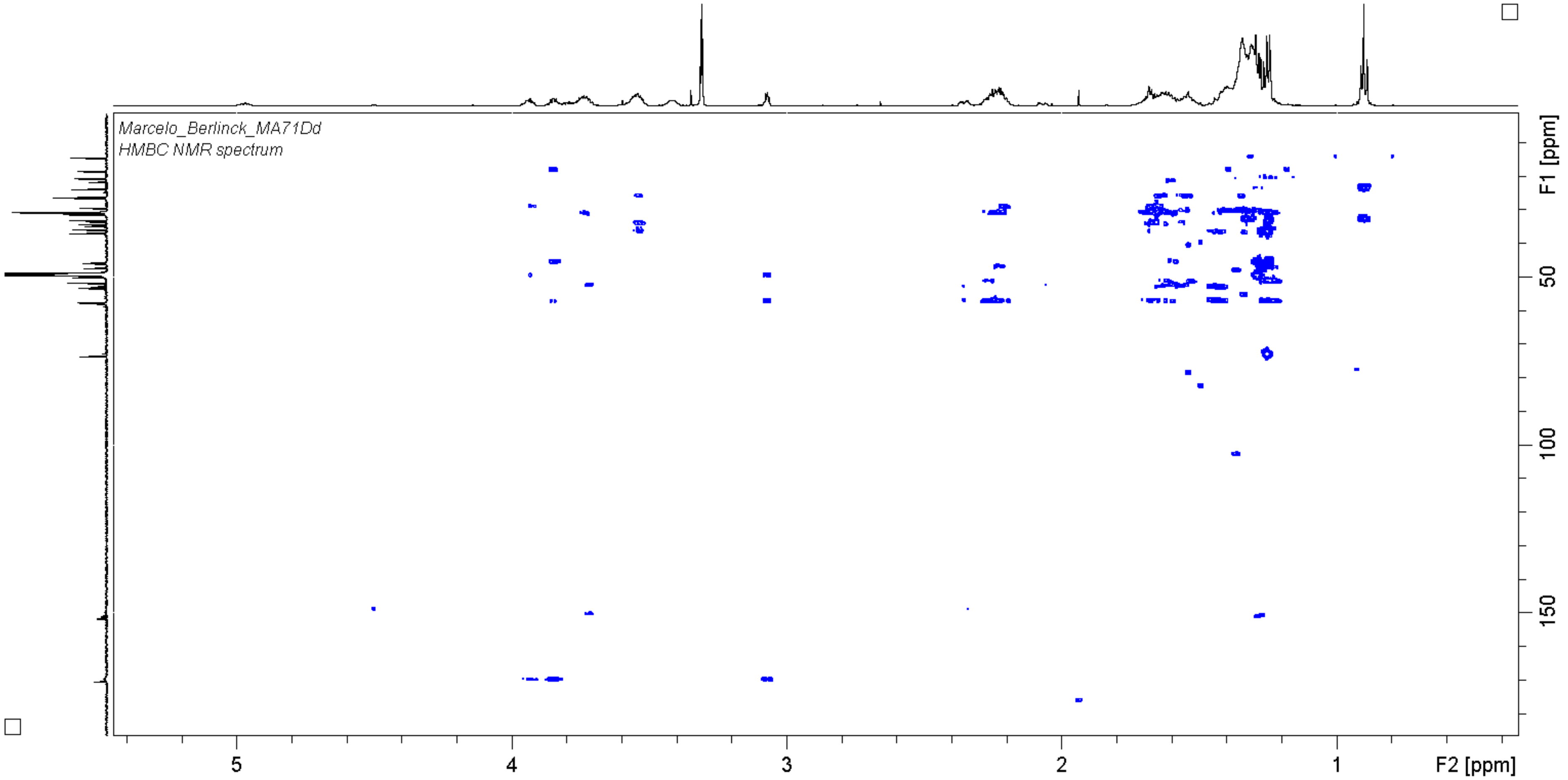

Figure S19. *g*HMBC NMR spectrum of **2** (^{1}H: 600 MHz, ^{13}C: 150 MHz, CD_3OD).

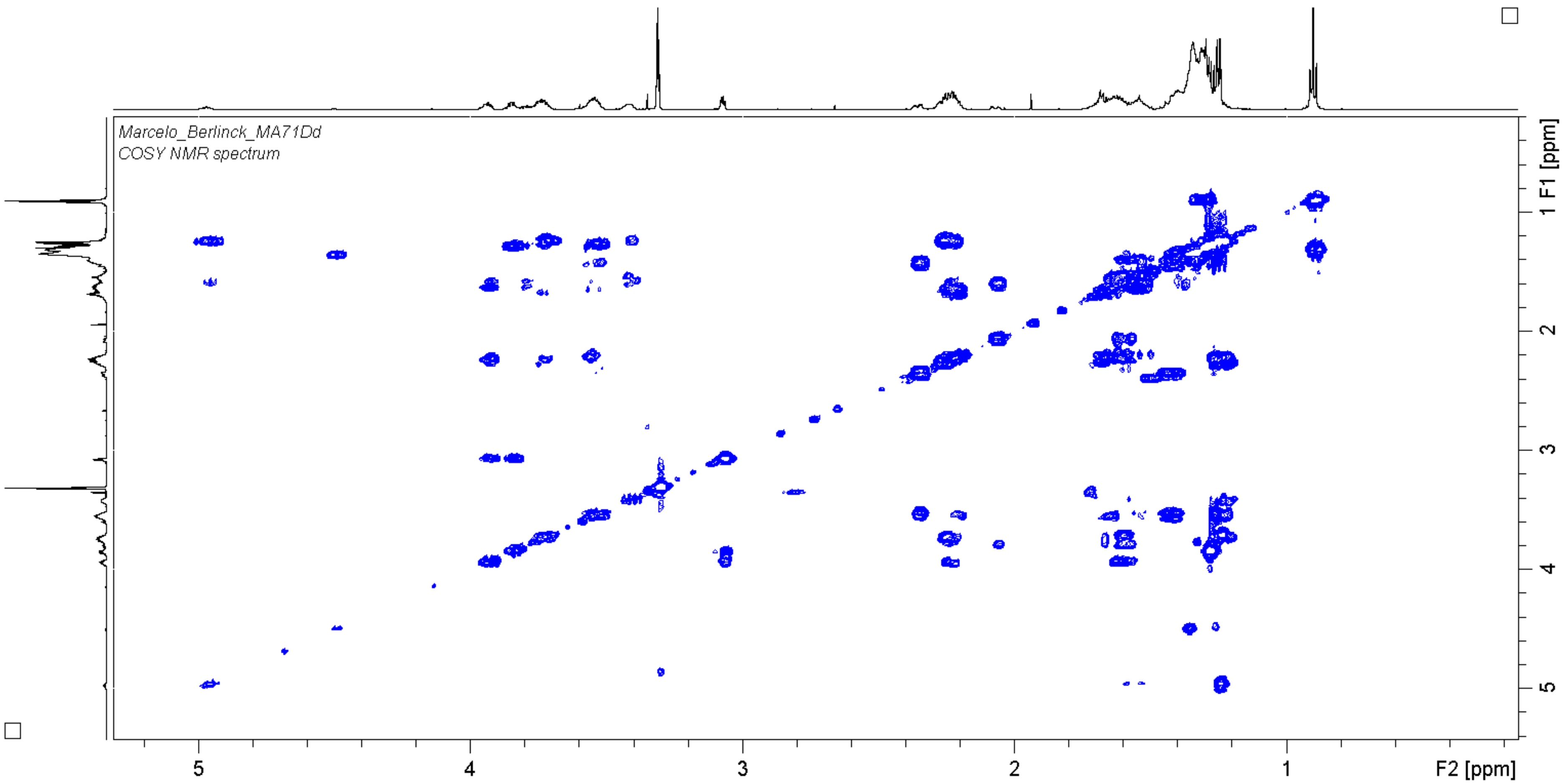

Figure S20. *g*COSY NMR spectrum of **2** (600 MHz, CD_3OD).

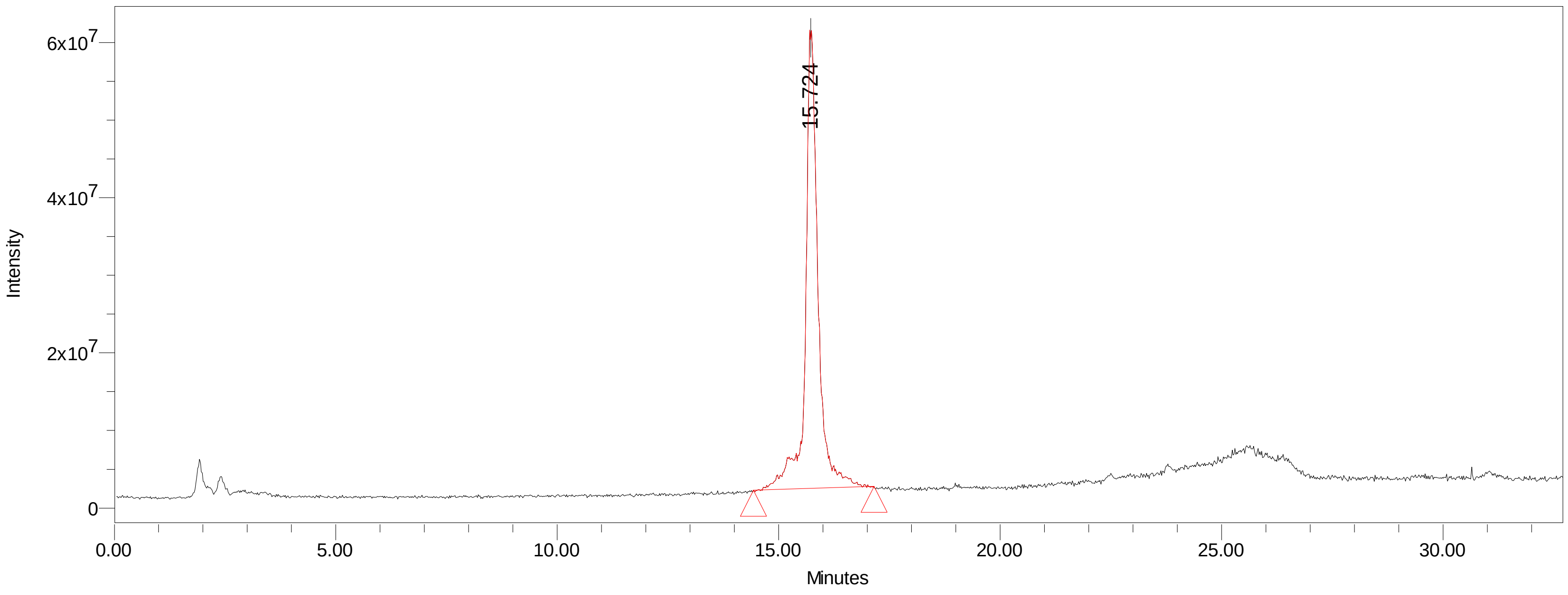

Figure S21. Total ion chromatogram (TIC) of **2** by HPLC-MS. Column C_{18} Xterra (Waters, 4.6 mm × 250 mm, 5 µm).

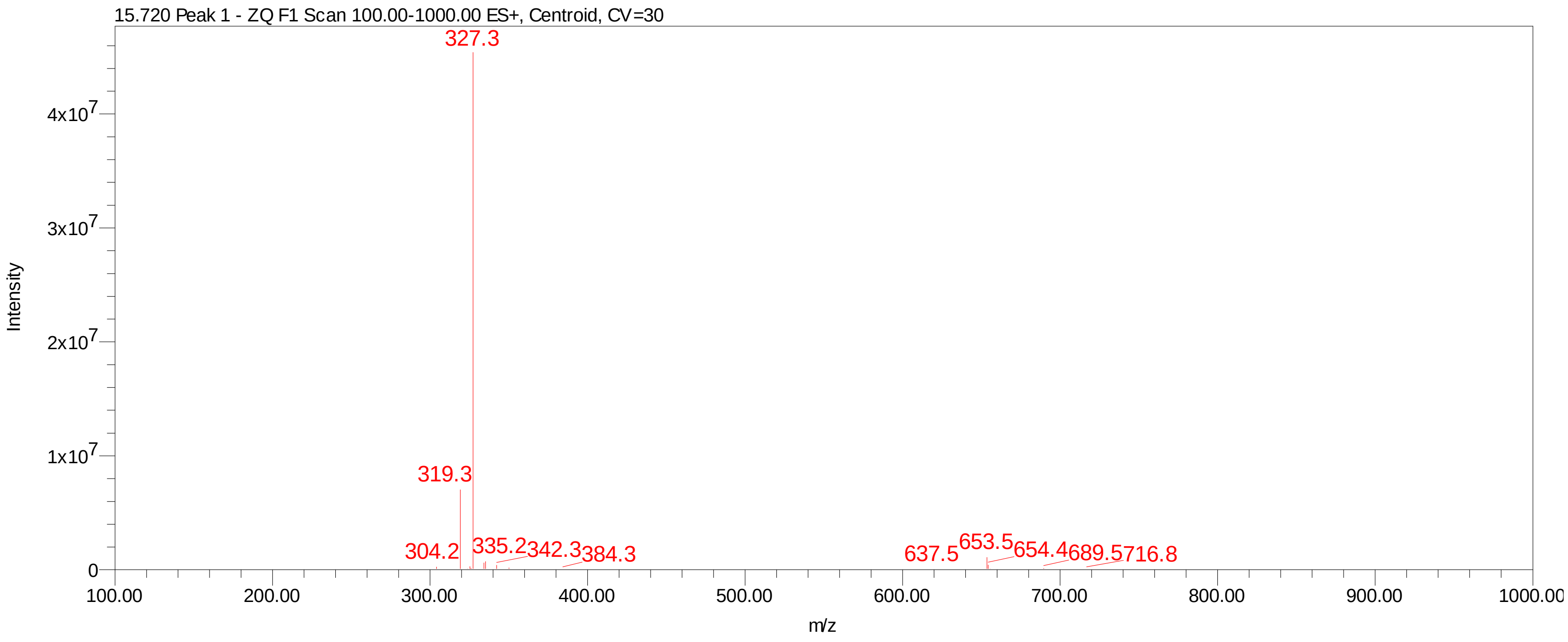

Figure S22. LR-ESI-MS spectrum of **2** in positive ionization mode by HPLC-MS.

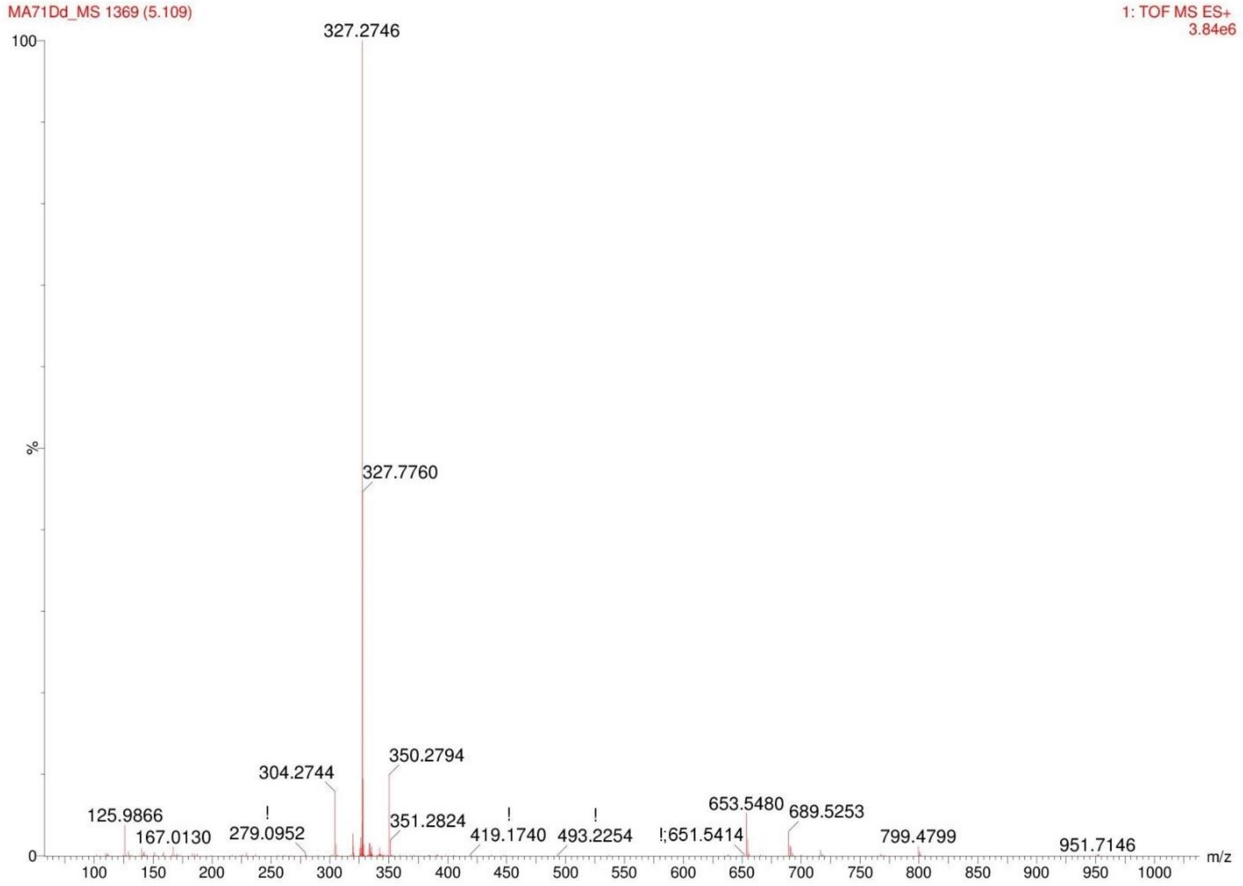

Figure S23. HR-ESI-MS spectrum of **2** in positive ionization mode by UPLC-ESI-qTOF-MS.

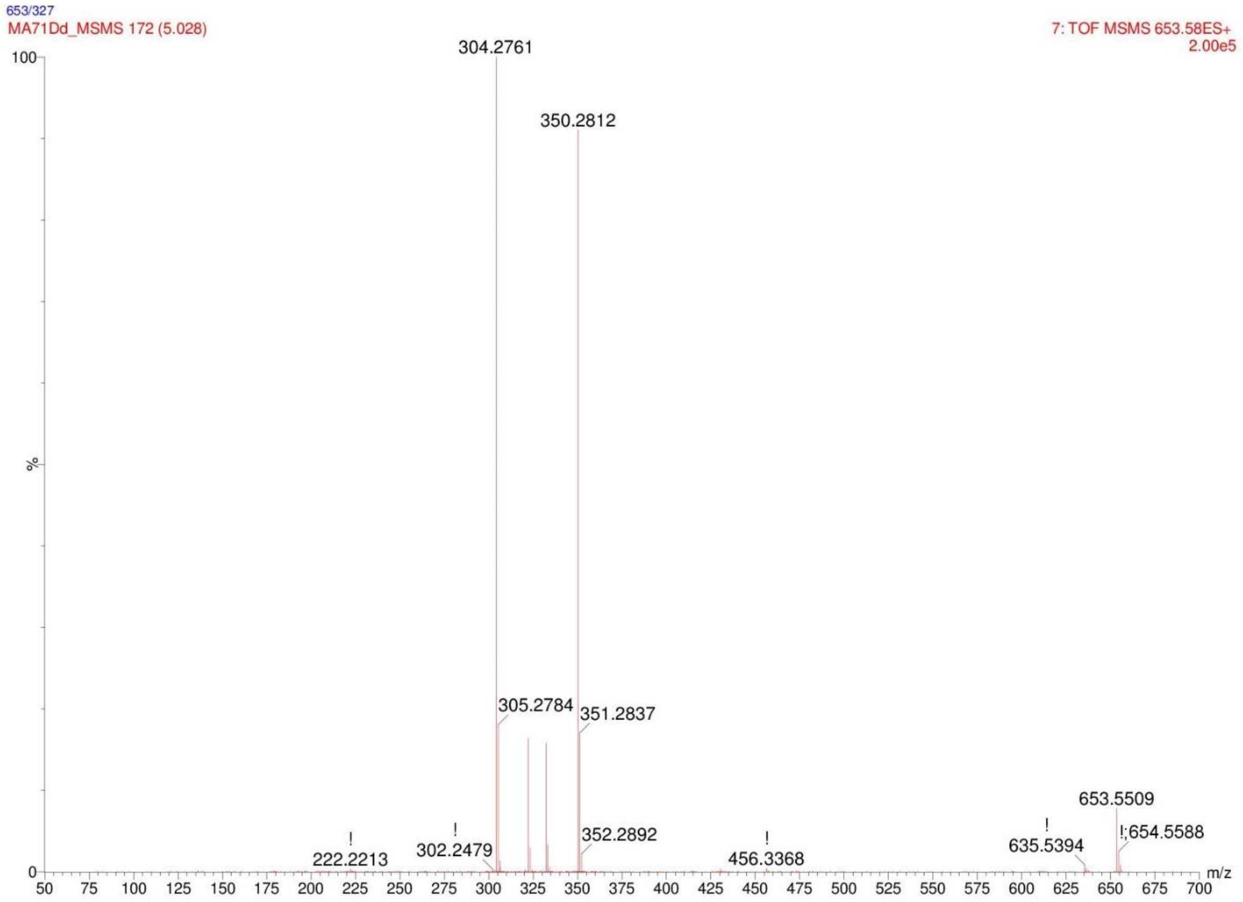

Figure S24. HR-ESI-qTOF-MS/MS spectrum of **2** in positive ionization mode.

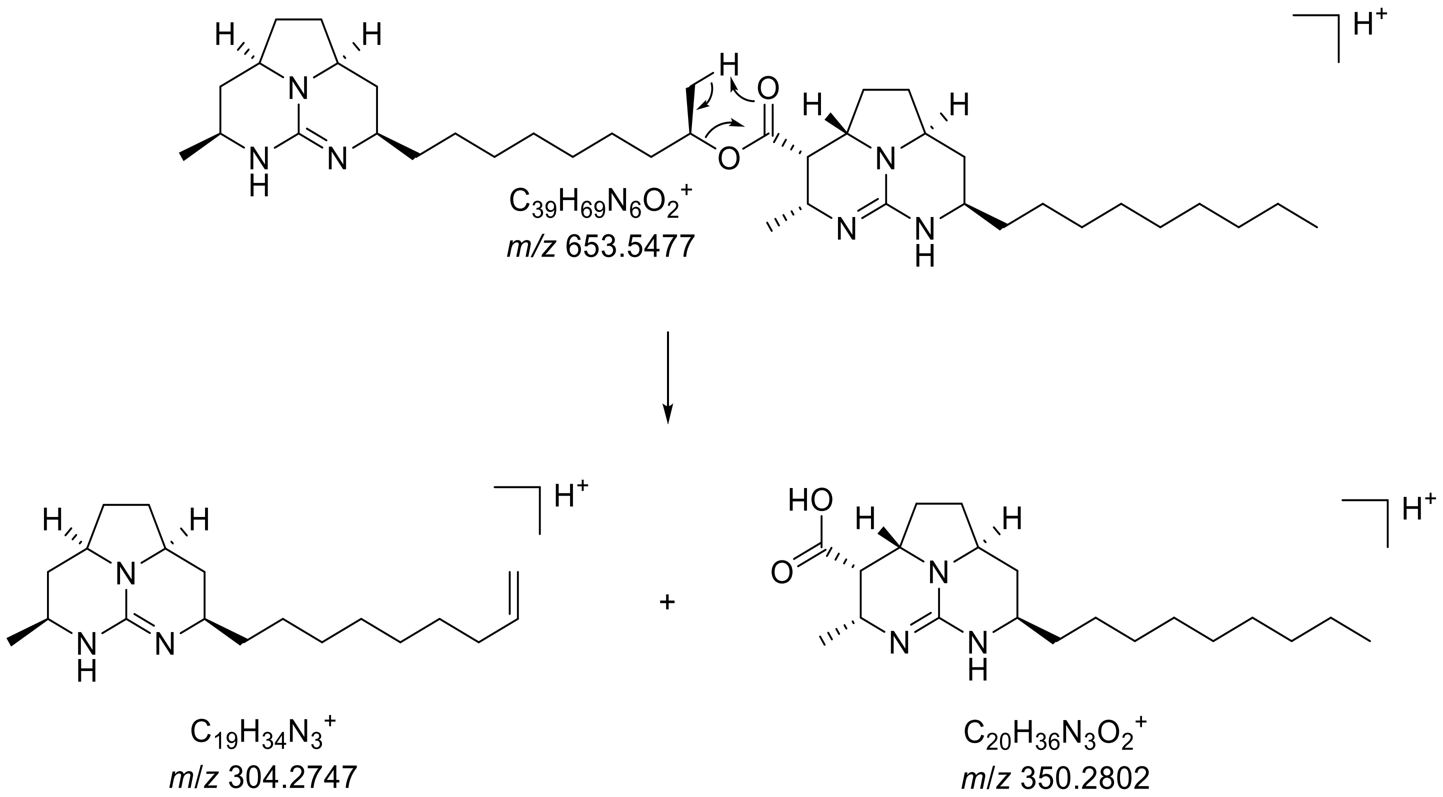

Figure S25. Fragmentation proposal for compound **2** by HR-ESI-qTOF-MS/MS in positive ionization mode.

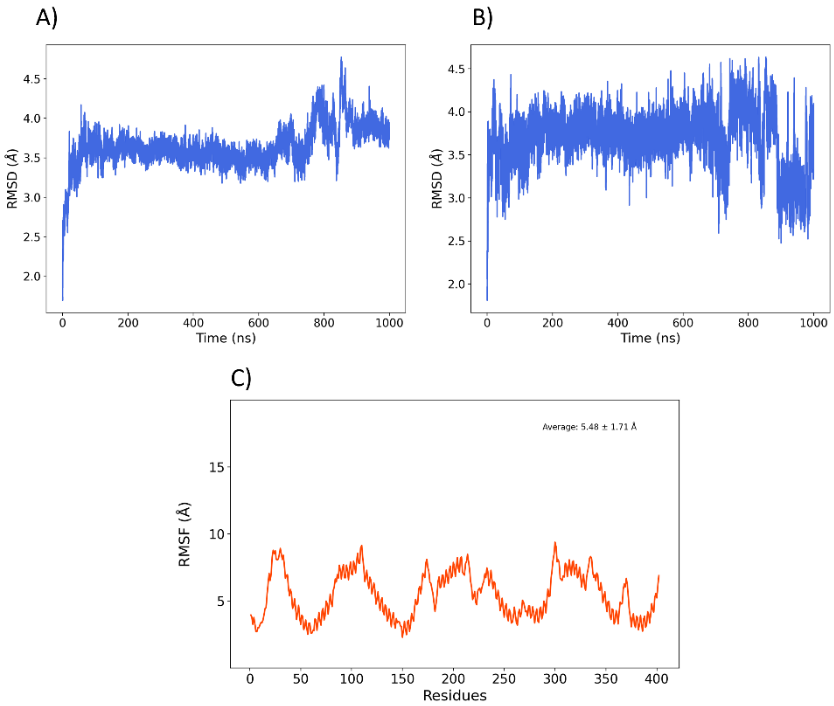

Figure S26. MD analysis of batzelladine F-*Pf*ENT1 over 1µs of simulation**. A)** RMSD of the protein-ligand complex; **B)** RMSD of the ligand; **C)** RMSF of the C_α atoms throughout the simulation.

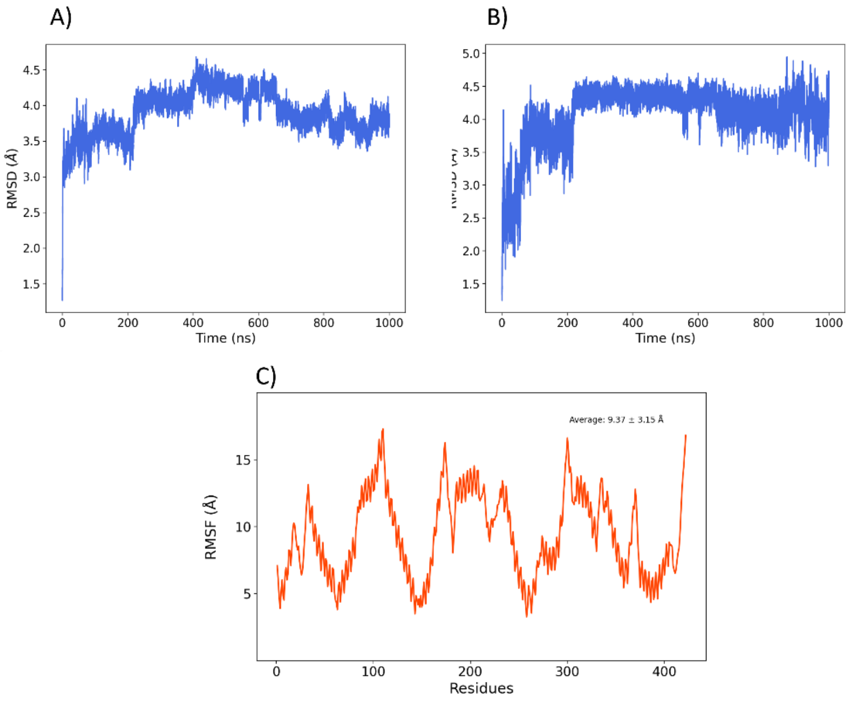

Figure S27. MD analysis of batzelladine L-*Pf*ENT1 over 1µs of simulation**. A)** RMSD of the protein-ligand complex; **B)** RMSD of the ligand; **C)** RMSF of the C_α atoms throughout the simulation.

For batzelladine F, the MD simulations confirmed a binding pose closely resembling the docking results. The protonated tricyclic guanidine moiety presented a new polar interaction with Glu618 for over 75% of the simulation time (Figure S28). Additionally, the ester group, initially interacting directly with Gln135 in the docking calculations, formed a stable water bridge between its carboxyl group and the side chain of Gln135 throughout the simulation. This water-mediated interaction was highly persistent (Figure S29) and appeared to be crucial for stabilizing batzelladine F in the binding pocket. In contrast, batzelladine L exhibited a slightly altered binding mode compared to the docking predictions. Over the course of the simulation, the compound shifted toward the intracellular environment, losing its direct interaction with Gln135. However, around 200 ns, the carboxyl group of batzelladine L established a stable interaction with Ser154, which persisted until the end of the simulation (Figure S30). batzelladine L also formed interactions with Glu618, similar to batzelladine F (Figure S31). The simulations suggest that batzelladine F forms more stable interactions with *Pf*ENT1, particularly maintaining the critical interaction with Gln135, which is consistent with its greater potency against the parasite in cellular assays compared to batzelladine L.

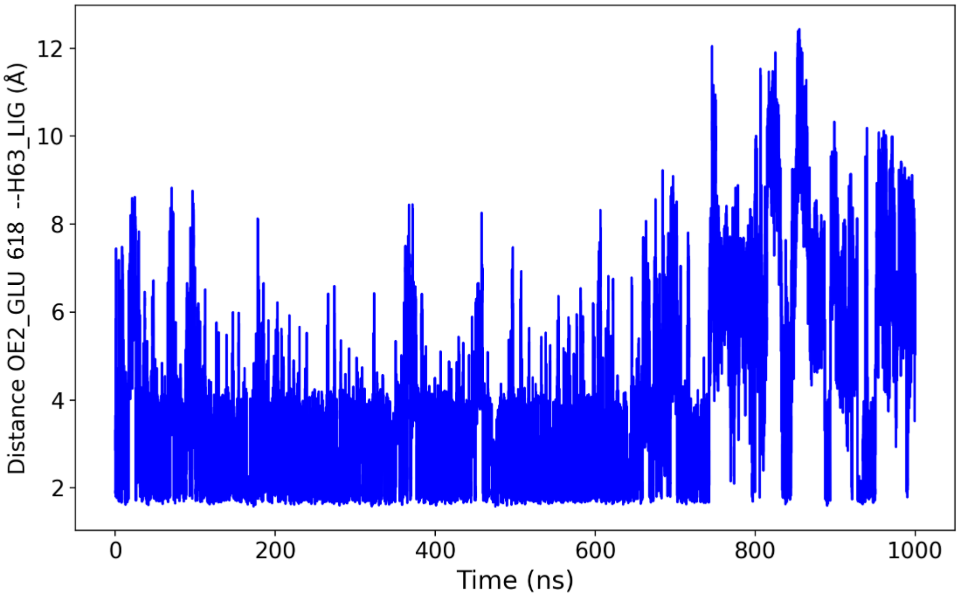

Figure S28. Distance between OE2 of Glu618 and H63 of the protonated tricyclic guanidine moiety in batzelladine F over 1μs of simulation.

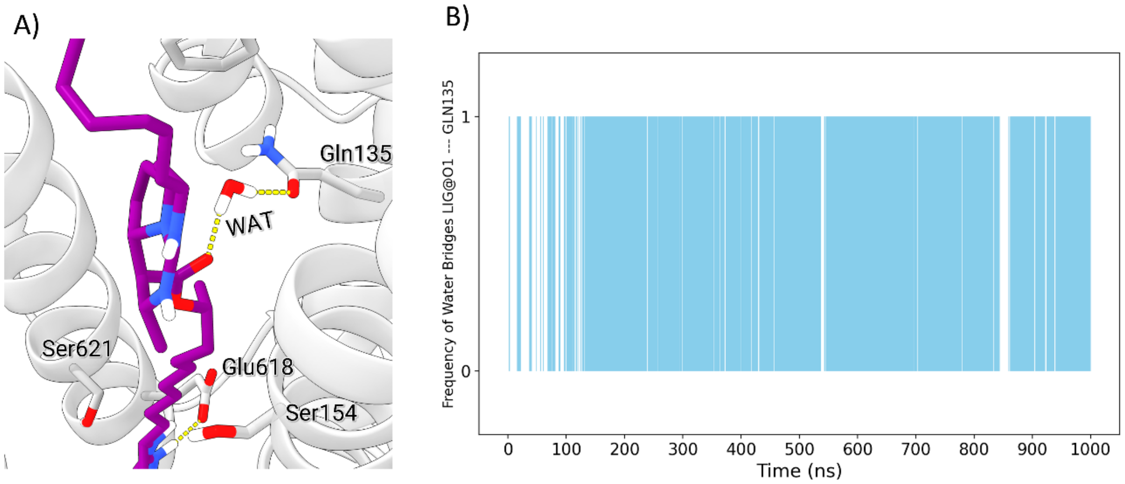

Figure S29. A) Binding mode of batzelladine F interacting with Gln135 through a water bridge. **B)** Frequency of the water bridge between the carbonyl O1 of batzelladine F and the side chain of Gln135 (C=O or NH_2) over 1μs of simulation. A value of 0 indicates no water bridge formation, while a value of 1 indicates the presence of a water bridge between batzelladine F and Gln135.

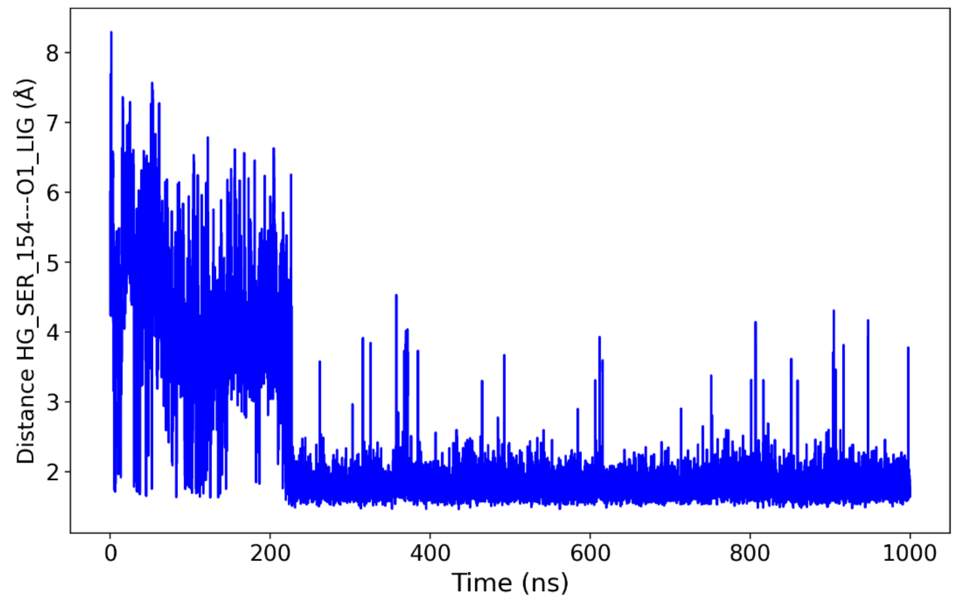

Figure S30. Distance between HG of Ser154 and the carbonyl O1 of batzelladine L over 1μs of simulation.

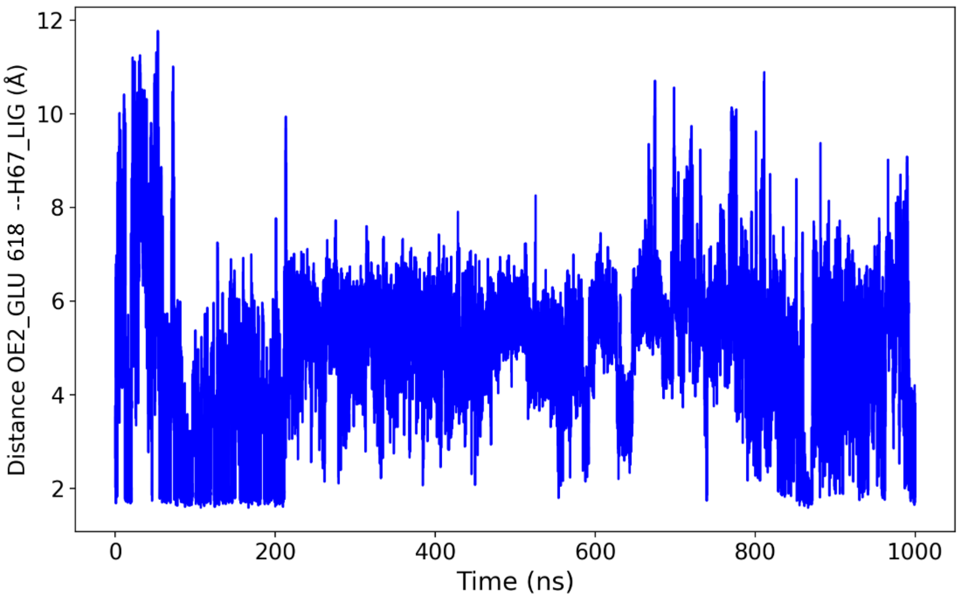

Figure S31. Distance between OE2 of Glu618 and H67 of the protonated tricyclic guanidine moiety in batzelladine L over 1μs of simulation.

Table S1. Comparison of ^{1}H (600 Hz) and ^{13}C (150 Hz) NMR data for **1** with the data reported in the literature for batzelladine F.

	**1**[1]		**Batzelladine F**[a]	
**No.**	**δ_H, mult. (*J* in Hz)**	**δ_C, type**	**δ_H, mult. (*J* in Hz)**	**δ_C, type**
1	1.25, d (6.3)	20.7, CH_3	1.26, d (6.3)	20.7, CH_3
2	3.55, m	47.3, CH	3.52, m	47.2, CH
3	2.25, m	36.8, CH_2	2.20, m	36.9, CH_2
	1.25, m		1.25, m	
4	3.73, m	57.6, CH	3.72, m	57.5, CH
5	2.22, m	31.1, CH_2	2.20, m	31.1, CH_2
	1.68, m		1.67, m	
6	2.22, m	31.1, CH_2	2.20, m	31.1, CH_2
	1.68, m		1.67, m	
7	3.73, m	57.5, CH	3.72, m	57.4, CH
8	2.26, m	34.8, CH_2	2.28, m	34.8, CH_2
	1.22, m		1.21, m	
9	3.40, m	51.6, CH	3.40, m	51.6, CH
10	-	150.6, C	-	151.2, C
11	1.63, m	35.9, CH_2	1.60, m	35.8, CH_2
	1.55, m		1.54, m	
12	1.30, m	26.2, CH_2	1.30, m	26.2, CH_2
13	1.30, m	30.3-30.7[b], CH_2	1.30, m	30.5, CH_2
14	1.30, m	26.6, CH_2	1.30, m	26.5, CH_2
15	1.60, m	36.9, CH_2	1.60, m	36.9, CH_2
	1.54, m		1.54, m	
16	4.97, m	73.4, CH	4.97, m	73.3, CH
17	1.25, d (6.1)	20.4, CH_3	1.24, d (6.5)	20.5, CH_3
18	-	170.4, C	-	170.3, C
19	3.08, dd (4.6, 3.3)	45.6, CH	3.06, dd (4.6, 3.3)	45.6, CH
20	3.95, m	58.0, CH	3.94, m	57.9, CH
21	2.25, m	29.2, CH_2	2.24, m	29.2, CH_2
	1.62, m		1.62, m	
22	2.21, m	31.4, CH_2	2.18, m	31.4, CH_2
	1.67, m		1.63, m	
23	3.56, m	57.3, CH	3.52, m	57.3, CH
24	2.35, m	34.2, CH_2	2.34, m	34.2, CH_2
	1.44, m		1.42, m	
25	3.54, m	53.2, CH	3.53, m	53.2, CH
26	-	151.2, C	-	151.6, C
27	3.84, m	49.9, CH	3.82, m	49.9, CH
28	1.27, d (6.7)	18.5, CH_3	1.27, d (6.7)	18.5, CH_3

	**1**[1]		**Batzelladine F**[a]	
**No.**	**δ_H, mult. (*J* in Hz)**	**δ_C, type**	**δ_H, mult. (*J* in Hz)**	**δ_C, type**
29	1.63, m	37.0, CH_2	1.64, m	36.9, CH_2
	1.55, m		1.54, m	
30	1.30, m	26.2, CH_2	1.33, m	26.2, CH_2
31	1.30, m	30.3-30.7[b], CH_2	1.30, m	30.5, CH_2
32	1.30, m	30.3-30.7[b], CH_2	1.30, m	30.5, CH_2
33	1.30, m	30.3-30.7[b], CH_2	1.30, m	30.5, CH_2
34	1.30, m	30.3-30.7[b], CH_2	1.30, m	30.5, CH_2
35	1.30, m	32.9, CH_2	1.30, m	33.0, CH_2
36	1.30, m	23.7, CH_2	1.30, m	23.7, CH_2
37	0.90, t (7.0)	14.4, CH_3	0.90, t (7.0)	14.5, CH_3

[a]A. D. Patil, A. J. Freyer, P. B. Taylor, B. Carté, G. Zuber, R. K. Johnson, D. J. Faulkner. Batzelladines F-I, Novel Alkaloids from the Sponge *Batzella* sp.: Inducers of p56[lck]-CD4 Dissociation. *J. Org. Chem.* **1997**, *62*, 1814-1819. [b]Carbon chemical shift between the range from 30.3 to 30.7. Overlapped correlations signals by HSQC spectrum.

Table S2. Comparison of ^1H (600 Hz) and ^{13}C (150 Hz) NMR data for **2** with the data reported in the literature for batzelladine L.

	**2**[1]		**Batzelladine L**[a]	
**No.**	**δ_H, mult. (J in Hz)**	**δ_C, type**	**δ_H, mult. (J in Hz)**	**δ_C, type**
1	1.27, d (6.4)	20.9, CH_3	1.26, d (6.4)	20.8, CH_3
2	3.54, m	47.4, CH	3.54, m	47.3, CH
3	2.22, m	37.0, CH_2	2.22, m	37.0, CH_2
	1.25, m		1.25, m	
4	3.74, m	57.8, CH	3.74, m	57.5, CH
5	2.22, m	31.2, CH_2	2.22, m	31.1, CH_2
	1.68, m		1.67, m	
6	2.22, m	31.2, CH_2	2.22, m	31.1, CH_2
	1.68, m		1.67, m	
7	3.74, m	57.7, CH	3.74, m	57.4, CH
8	2.26, m	34.9, CH_2	2.24, m	34.8, CH_2
	1.24, m		1.23, m	
9	3.42, m	51.7, CH	3.41, m	51.6, CH
10	-	151.3, C	-	151.1, C
11	1.62, m	35.9, CH_2	1.60, m	35.9, CH_2
	1.53, m		1.48, m	
12	1.34, m	26.3, CH_2	1.33, m	26.2, CH_2
13	1.34, m	30.5 - 30.7[b], CH_2	1.33, m	30.5, CH_2
14	1.34, m	30.5 - 30.7[b], CH_2	1.33, m	30.5, CH_2
15	1.34, m	30.5 - 30.7[b], CH_2	1.33, m	30.5, CH_2
16	1.34, m	26.7, CH_2	1.33, m	26.5, CH_2
17	1.39, m	37.0, CH_2	1.33, m	36.8, CH_2
18	4.96, m	73.5, CH	4.97, m	73.4, CH
19	1.25, d (6.3)	20.6, CH_3	1.24, d (6.4)	20.5, CH_3
20	-	170.4, C	-	170.4, C
21	3.07, dd (4.5, 3.5)	46.0, CH	3.08, t (4.0)	45.6, CH
22	3.93, m	57.8, CH	3.96, m	57.9, CH
23	2.23, m	29.5, CH_2	2.23, m	29.2, CH_2
	1.62, m		1.62, m	
24	2.22, m	31.6, CH_2	2.20, m	31.4, CH_2
	1.64, m		1.64, m	
25	3.53, m	53.4, CH	3.54, m	57.3, CH
26	2.36, m	34.3, CH_2	2.34, m	34.2, CH_2
	1.43, m		1.42, m	

	**2**[1]		**Batzelladine L**[a]	
**No.**	**δ_H, mult. (*J* in Hz)**	**δ_C, type**	**δ_H, mult. (*J* in Hz)**	**δ_C, type**
27	3.56, m	57.6, CH	3.54, m	53.2, CH
28	-	151.7, C	-	151.5, C
29	3.85, m	50.0, CH	3.84 (m)	49.9, CH
30	1.29, d (6.7)	18.6, CH_3	1.28, d (6.4)	18.6, CH_3
31	1.63, m	37.1, CH_2	1.64, m	37.0, CH_2
	1.55, m		1.54, m	
32	1.34, m	26.3, CH_2	1.33, m	26.2, CH_2
33	1.34, m	30.5-30.7[b], CH_2	1.33, m	30.5, CH_2
34	1.34, m	30.5-30.7[b], CH_2	1.33, m	30.5, CH_2
35	1.34, m	30.5-30.7[b], CH_2	1.33, m	30.5, CH_2
36	1.34, m	30.5-30.7[b], CH_2	1.33, m	30.5, CH_2
37	1.30, m	33.2, CH_2	1.33, m	33.0, CH_2
38	1.32, m	23.9, CH_2	1.33, m	23.7, CH_2
39	0.90, t (7.0)	14.6, CH_3	0.90, t (7.0)	14.5, CH_3

[a]H.-M. Hua, J. Peng, D. C. Dunbar, R. F. Schinazi, A. G. de C. Andrews, C. Cuevas, L. F. Garcia-Fernandez, M. Kelly, M. T. Hamann. Batzelladine alkaloids from the caribbean sponge *Monanchora unguifera* and the significant activities against HIV-1 and AIDS opportunistic infectious pathogens. *Tetrahedron*, **2007**, *63*, 11179–11188.
[b]Carbon chemical shift between the range from 30.5 to 30.7. Overlapped correlations signals by HSQC spectrum.

Molecular Modelling Procedures

Molecular docking calculations

The *Pf*ENT1 structure was obtained though the PDB code: 7YDQ[2] and the protonate states of the amino acids were assigned with PDB2PQR[3] at the same pH as the reported biochemical assays. For the ligands, we checked their p*K*a with MolGpKa.[4] The possible tautomeric forms of the batzelladines with both guanidine moieties protonated were then accessed through Quacpac.[5] Mol2 files of the ligands were generated using Omega.[6] Next, both compounds were docked using GOLD[7], with the binding pocket defined as all atoms within 8 Å away from the ligand in the PDB 7YDQ. Pose analysis involved visual inspection for hydrogen bond formation with Gln135 and comparison of the positioning of the carbonyl group involved in this interaction with the bound ligand.

Molecular dynamics simulations

To complete the structure of *Pf*ENT1 (7YDQ) with the missing residues and atoms, we employed AlphaFold2,[8] using the PDB itself as a template to guide the structure prediction. The protonation state of the amino acids in the complete *Pf*ENT1 structure was performed in the PDB2PQR server.[3] The full structure was then aligned with the protein-ligand complexes of BatL and BatF, obtained from docking calculations, to position the ligands correctly within the transporter.

Each protein-ligand complex was embedded in an explicit lipid bilayer using the CHARMM-GUI Membrane Builder.[9–11] The membrane composition included POPC, POPE, POPS and cholesterol in a 25:5:5:1 ratio, following Zhang's work for *Pf*NCR1 parasite plasma membrane.[12] A 150 mM NaCl concentration was added to neutralize the systems. Finally, the solvated *Pf*ENT1-BatL complex contained 135,109 atoms, while the *Pf*ENT1-BatF complex contained 135,262 atoms.

The atomic charges of the ligands were derived using AM1-BCC in antechamber and parameters were assigned using GAFF2. The force field ff14SB was employed for the protein, Lipid21 for the lipids and the TIP3P was used as the water model. Each system was subjected to an initial minimization of 10,000 steps, followed by gradual heating from 0 K to 303.15 K in the NVT ensemble using the Langevin thermostat. Next, an NPT equilibration was performed at 303.15 K, followed by a 1 µs production run with atomic coordinates of each system saved every 50 ps for analysis. The all-atom MD simulations were performed using Amber24 with pmemd.cuda.[13] Trajectory analyses, including hydrogen bond interactions, water bridge occurrences, RMSD, and RMSF calculations, were conducted using cpptraj[14] and MDAnalysis.[15,16] Representative conformations of each system were obtained through k-means clustering into 10 clusters over 500 iterations based on the ligand-protein RMSD, excluding hydrogen atoms.

General Experimental Procedures

Optical rotations were measured on a Jasco P-2000 polarimeter. NMR experiments were recorded on a Bruker Avance III instrument (^{1}H: 600 MHz; ^{13}C: 150 MHz) with an inverse cryoprobe of 5 mm with z-field gradient. The chemical shifts (δ) were expressed in ppm and recorded with reference to solvent signals (^{1}H NMR: MeOH-d_4 3.31 ppm; ^{13}C NMR: MeOH-d_4 49.0 ppm). MeOH-d_4 NMR solvent was purchased from Cambridge Isotope Laboratories. The analytical HPLC-PDA-MS system was a Waters instrument (2695 Alliance separation module, 2696 photodiode array detector) coupled to a Waters Micromass ZQ 2000 mass spectrometer. Analyses were performed using a Waters XTerra RP18 column (250 mm × 4.6 mm, 5 µm) along with the RP18 protective guard column (4 × 3 mm) and eluting with H_2O + 0.1% formic acid, MeOH + 0.1% formic acid, and

MeCN + 0.1% formic acid using a gradient from 90:5:5 to 0:50:50 of H_2O/MeOH/MeCN over 22 min, maintaining in 0:50:50 H_2O/MeOH/MeCN for 8 min, from 0:50:50 to 90:5:5 in 1 min, and maintaining at 90:5:5 for 9 min, using a flow rate of 1.0 mL/min. The PDA detector scanned between λ 200 and 600 nm. The mass spectrometer detector was optimized using the following conditions: capillary voltage 3 kV; temperature of the source 100 °C; desolvation temperature 350 °C; ESI mode, acquisition range 100 to 1200 Da; gas flow without cone 50 L/h; desolvation gas flow 350 L/h. Samples were diluted in MeOH at a concentration of 1 mg/mL. UPLC-ESI-qTOF-MS were acquired on a Waters Acquity H-Class UPLC coupled to a Xevo G2-XS Q-TOF instrument with an electrospray ionization (ESI) interface in the positive ionization mode. Acquisition time of 0 to 10 minutes, mass range of 50 to 1200 Da, scan time of 0.2 s^{-1}. The positive mode ESI conditions were: 1.2 kV capillary voltage, 30 V cone voltage, 100 °C source temperature, desolvation temperature of 450 °C, 50 L h^{-1} cone gas flow and 750 L h^{-1} desolvation gas flow. For internal calibration, a solution of leucine enkephalin (Sigma) 200 pg mL^{-1}, was infused by the lock-mass probe with a flow rate of 10 μL min^{-1} was used. Chromatographic separations were performed using a BEH C_{18} column (2.1 × 100 mm, 1.7 μm, Waters) and a mobile phase consisting of Milli-Q + 0.1% formic acid and MeCN + 0.1% formic acid. The elution gradient used was from 90:10 of H_2O/MeCN to 50:50 H_2O/MeCN for 6 min, 50:50 of H_2O/MeCN to 2:98 H_2O/MeCN for 3 min, 2:98 of H_2O/MeCN to 90:10 H_2O/MeCN for 0.1 min and H_2O/MeCN 90:10 for 0.9 min, flow rate of 0.50 mL min^{-1}. The column was maintained at a temperature of 40 °C and the samples were maintained at 15 °C. Samples were diluted in MeOH at a concentration of 0.01 mg mL^{-1}. Semipreparative HPLC separations were performed on a Waters system, including a Waters 600 quaternary pump and a Waters 2996 controller coupled to a Waters 2487 dual absorbance detector.

References

(1) Pedras, M. S. O. C.; Smith, K. C.; Taylor, J. L. Production of 2,5-Dioxopiperazine by a New Isolate Type of the Blackleg Fungus Phoma Lingam. *Phytochemistry* 1998, *49* (6), 1575–1577.

(2) Wang, C.; Yu, L.; Zhang, J.; Zhou, Y.; Sun, B.; Xiao, Q.; Zhang, M.; Liu, H.; Li, J.; Li, J.; Luo, Y.; Xu, J.; Lian, Z.; Lin, J.; Wang, X.; Zhang, P.; Guo, L.; Ren, R.; Deng, D. Structural Basis of the Substrate Recognition and Inhibition Mechanism of Plasmodium Falciparum Nucleoside Transporter PfENT1. *Nat Commun* 2023, *14* (1). https://doi.org/10.1038/s41467-023-37411-1.

(3) Jurrus, E.; Engel, D.; Star, K.; Monson, K.; Brandi, J.; Felberg, L. E.; Brookes, D. H.; Wilson, L.; Chen, J.; Liles, K.; Chun, M.; Li, P.; Gohara, D. W.; Dolinsky, T.; Konecny, R.; Koes, D. R.; Nielsen, J. E.; Head-Gordon, T.; Geng, W.; Krasny, R.; Wei, G. W.; Holst, M. J.; McCammon, J. A.; Baker, N. A. Improvements to the APBS Biomolecular Solvation Software Suite. *Protein Science* 2018, *27* (1), 112–128. https://doi.org/10.1002/pro.3280.

(4) Pan, X.; Wang, H.; Li, C.; Zhang, J. Z. H.; Ji, C. MolGpka: A Web Server for Small Molecule PKaPrediction Using a Graph-Convolutional Neural Network. *J Chem Inf Model* 2021, *61* (7), 3159–3165. https://doi.org/10.1021/acs.jcim.1c00075.

(5) QUACPAC 2.2.3.3. OpenEye, Cadence Molecular Sciences, Santa Fe, NM.

(6) Hawkins, P. C. D.; Skillman, A. G.; Warren, G. L.; Ellingson, B. A.; Stahl, M. T. Conformer Generation with OMEGA: Algorithm and Validation Using High Quality Structures from the Protein Databank and Cambridge Structural Database. *J Chem Inf Model* 2010, *50* (4). https://doi.org/10.1021/ci100031x.

(7) Jones, G.; Willett, P.; Glen, R. C.; Leach, A. R.; Taylor, R. *Development and Validation of a Genetic Algorithm for Flexible Docking*.

(8) Jumper, J.; Evans, R.; Pritzel, A.; Green, T.; Figurnov, M.; Ronneberger, O.; Tunyasuvunakool, K.; Bates, R.; Žídek, A.; Potapenko, A.; Bridgland, A.; Meyer, C.; Kohl, S. A. A.; Ballard, A. J.; Cowie, A.; Romera-Paredes, B.; Nikolov, S.; Jain, R.; Adler, J.; Back, T.; Petersen, S.; Reiman, D.; Clancy, E.; Zielinski, M.; Steinegger, M.; Pacholska, M.; Berghammer, T.; Bodenstein, S.; Silver, D.; Vinyals, O.; Senior, A. W.; Kavukcuoglu, K.; Kohli, P.; Hassabis, D. Highly Accurate Protein Structure Prediction with AlphaFold. *Nature* 2021, *596* (7873), 583–589. https://doi.org/10.1038/s41586-021-03819-2.

(9) Jo, S.; Kim, T.; Iyer, V. G.; Im, W. CHARMM-GUI: A Web-Based Graphical User Interface for CHARMM. *J Comput Chem* 2008, *29* (11), 1859–1865. https://doi.org/10.1002/jcc.20945.

(10) Wu, E. L.; Cheng, X.; Jo, S.; Rui, H.; Song, K. C.; Dávila-Contreras, E. M.; Qi, Y.; Lee, J.; Monje-Galvan, V.; Venable, R. M.; Klauda, J. B.; Im, W. CHARMM-GUI Membrane Builder toward Realistic Biological Membrane Simulations. *Journal of Computational Chemistry*. John Wiley and Sons Inc. October 15, 2014, pp 1997–2004. https://doi.org/10.1002/jcc.23702.

(11) Jo, S.; Lim, J. B.; Klauda, J. B.; Im, W. CHARMM-GUI Membrane Builder for Mixed Bilayers and Its Application to Yeast Membranes. *Biophys J* 2009, *97* (1), 50–58. https://doi.org/10.1016/j.bpj.2009.04.013.

(12) Zhang, Z.; Lyu, M.; Han, X.; Bandara, S.; Cui, M.; Istvan, E. S.; Geng, X.; Tringides, M. L.; Gregor, W. D.; Miyagi, M.; Oberstaller, J.; Adams, J. H.; Zhang, Y.; Nieman, M. T.; von Lintig, J.; Goldberg, D. E.; Yu, E. W. The Plasmodium Falciparum NCR1 Membrane Protein Is a Novel Antimalarial Target That Exports Cholesterol to Maintain Membrane Homeostasis. September 30, 2024. https://doi.org/10.1101/2024.09.30.615907.

(13) D.A. Case et al. Amber 2024. University of California, San Francisco 2024.

(14) Roe, D. R.; Cheatham, T. E. PTRAJ and CPPTRAJ: Software for Processing and Analysis of Molecular Dynamics Trajectory Data. *J Chem Theory Comput* 2013, *9* (7), 3084–3095. https://doi.org/10.1021/ct400341p.

(15) Michaud-Agrawal, N.; Denning, E. J.; Woolf, T. B.; Beckstein, O. MDAnalysis: A Toolkit for the Analysis of Molecular Dynamics Simulations. *J Comput Chem* 2011, *32* (10), 2319–2327. https://doi.org/10.1002/jcc.21787.

(16) Gowers, R. J.; Linke, M.; Barnoud, J.; E Reddy, T. J.; Melo, M. N.; Seyler, S. L.; Domá nski, J.; Dotson, D. L.; Buchoux, S.; Kenney, I. M.; Beckstein, O. *MDAnalysis: A Python Package for the Rapid Analysis of Molecular Dynamics Simulations*; 2016. http://mdanalysis.org.
